# *Legionella pneumophila* modulates the host cytoskeleton by an effector of transglutaminase activity

**DOI:** 10.1002/mlf2.70013

**Published:** 2025-06-18

**Authors:** Yan Liu, Yao Liu, Zhao‐Qing Luo

**Affiliations:** ^1^ Department of Biological Sciences, Purdue Institute for Inflammation, Immunology and Infectious Diseases Purdue University West Lafayette Indiana USA; ^2^ Present address: Department of Biological Sciences University of Notre Dame South Bend IN USA

**Keywords:** Dot/Icm type IV secretion system, *Legionella pneumophila*, metaeffector LegL1, transglutaminase activity

## Abstract

The bacterial pathogen *Legionella pneumophila* delivers more than 330 effector proteins into host cells through its Dot/Icm type IV secretion system (T4SS) to facilitate its intracellular replication. A number of these effectors modulate organelle trafficking pathways to create a membrane‐bound niche called the *Legionella*‐containing vacuole (LCV). In this study, we found that *L. pneumophila* induces F‐actin accumulation in the host cell cortex by its Dot/Icm substrate RavJ (Lpg0944). RavJ harbors a C_101_H_138_D_170_ motif associated with human tissue transglutaminases (TGs). We show that RavJ catalyzes a covalent linkage between actin and members of the Motin family of proteins, including Angiomotin (AMOT) and Angiomotin‐like 1 (AMOTL1), which are known to regulate cell migration and contribute to the formation of cellular structures such as endothelial cell junctions and tubes. Further study reveals that RavJ‐induced crosslink between actin and AMOT occurs on its Gln_354_ residue. Crosslink between actin and AMOT significantly reduces the binding between actin and its binding partner cofilin, suggesting that RavJ inhibits actin depolymerization. We also demonstrate that the metaeffector LegL1 directly interacts with RavJ to antagonize its TG activity, leading to reduced crosslinks between actin and Motin proteins. Our results reveal a novel mechanism of modulating the host actin cytoskeleton by *L. pneumophila*.

## Impact statement

This study uncovers a novel mechanism by which *Legionella pneumophila* manipulates host cell actin dynamics. We show that the bacterial effector RavJ catalyzes a covalent crosslink between actin and the Angiomotin (AMOT) family of proteins, which are crucial for cell migration and cytoskeleton structural organization. RavJ‐induced crosslink impacts actin turnover to alter host cell behavior for the creation of a niche permissive for bacterial replication. The identification of RavJ as a transglutaminase (TG) has provided new insights into how *Legionella* exploits host cell machinery, which may facilitate functional studies of other TGs and offer potential avenues for therapeutic intervention.

## INTRODUCTION

*Legionella pneumophila* is a Gram‐negative intracellular pathogen that causes Legionnaires’ disease in humans^1^. Successful colonization by this bacteria requires its ability to manipulate diverse processes of host cells such as membrane trafficking, immunity, protein translation, autophagy, gene expression, and cytoskeleton structure^2^, ^3^. Upon entry into host cells, *L. pneumophila* promotes the biogenesis of a phagosome structure called the *Legionella*‐containing vacuole (LCV) that supports its intracellular replication^4^. The virulence of *L. pneumophila* is correlated with its ability to survive and replicate in the LCV^4^. Biogenesis of the LCV requires the Dot/Icm system that transports over 330 protein substrates into host cells^5^, ^6^, ^7^. The activity of these effectors is essential for the development and maintenance of this replicative niche^4^.

The actin cytoskeleton plays a critical role in numerous cellular processes, including mitosis, cell migration, epithelial barrier regulation, and immune cell adhesion^8^. Given its central importance, it is frequently targeted by bacterial virulence factors^9^. *Yersinia* spp. blocks macrophage phagocytosis by interfering with host Rho GTPase and the actin cytoskeleton dynamics^10^. *Salmonella enterica* Typhimurium delivers a subset of bacterial effector proteins into host cells to modulate the host cell actin cytoskeleton, facilitating bacterial entry into non‐phagocytic cells^11^. *L. pneumophila* has also been shown to modulate the actin cytoskeleton by its Dot/Icm substrates. For example, RavK is a protease that disrupts host cytoskeletal structure by cleaving actin^12^; LegK2 targets the actin nucleator ARP2/3 complex by phosphorylating its components ARPC1B and ARP3, leading to the global actin cytoskeleton remodeling in cells^13^; Ceg14 affects actin distribution and inhibits actin polymerization by a yet unknown mechanism^14^; and VipA interferes with organelle trafficking by acting as a nucleator for actin polymerization^15^. MavH regulates actin dynamics by promoting membrane tubulation through phosphatidylinositol 3‐phosphate (PI(3)P)‐dependent actin polymerization and recruitment of actin capping protein, which facilitates early stages of *L. pneumophila* infection^16^.

Induction of posttranslational modifications (PTMs) on host proteins involved in important cellular processes is a common mechanism used by bacterial pathogens to counteract host defense^17^. Such PTMs are often executed by virulence factors that display diverse biochemical activities. A number of PTMs have been found to be imposed by Dot/Icm effectors, including phosphorylcholination^18^, ^19^, AMPylation^20^, phosphorylation^21^, ADP‐ribosylation^22^, ^23^, ^24^, ubiquitination^22^, ^25^, and protein crosslinking mediated by transglutaminase (TG) activity^25^. Among these modifications, TGs catalyze the formation of isopeptide bonds by linking the γ‐carboxamide group of a glutamine residue on one protein to the ε‐amino group of a lysine residue on another, accompanied by the release of ammonia^26^. This modification has been identified as an important PTM that attacks a wide spectrum of host functions to benefit the pathogens. For example, the HopX (AvrPphE) family of *Pseudomonas syringae* Type III effectors contains a conserved cysteine‐base catalytic triad resembling that of TGs, which is required to trigger a cell‐death response in specific *Arabidopsis* ecotypes^27^. The type III effector VopC promotes *Vibrio* spp. invasion by activating Rac and CDC42 via its TG activity^28^. Transglutamination‐induced modifications have recently emerged as an important mechanism of virulence by *L. pneumophila*. The bacterial TG MavC catalyzes atypical ubiquitination reaction by crosslinking ubiquitin to the E2 enzyme UBE2N, leading to the inhibition of NF‐κB signaling in the initial phase of bacterial infection^25^.

Here, we show that the Dot/Icm substrate RavJ (Lpg0944) catalyzes a crosslink between actin and two members of the Motin family, Angiomotin (AMOT) and Angiomotin‐like 1 (AMOTL1), via its TG activity, leading to the accumulation of actin polymers in mammalian cells. We also show that the TG activity of RavJ is regulated by another Dot/Icm substrate LegL1(Lpg0945), which directly binds to RavJ and inhibits its enzymatic activity.

## RESULTS

### RavJ is a TG that induces actin accumulation in mammalian cells

**Figure 1:**
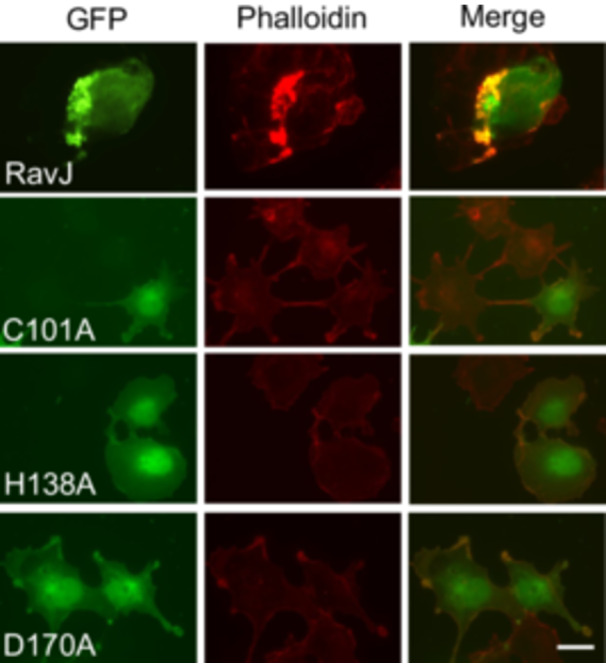
Ectopic expression of RavJ causes rearrangement of the actin cytoskeleton. HEK293T cells were transfected with the indicated constructs and then subjected to immunofluorescence microscopic analysis. F‐actin was stained by phalloidin conjugated with Texas‐Red. Bar, 10 µm. Note that wild‐type RavJ induces actin accumulation in the cell cortex and this phenotype is dependent on the predicted transglutaminase (TG) enzymatic motif.

RavJ was originally identified in a study aiming to analyze the mechanism of metaeffector activity in *L. pneumophila* ^29^. Structural analysis revealed that RavJ harbors a C‐H‐D (C_101_H_138_D_170_) motif associated with members of tissue TGs^29^. In a screening to identify Dot/Icm substrates capable of regulating the actin cytoskeleton, we transfected HEK293T cells with a GFP fusion library of Dot/Icm substrates and found that ectopically expressed RavJ caused rearrangements of the actin cytoskeleton (Figure 1). To determine the role of the putative catalytic motif potentially involved in TG activity in the function of RavJ, we introduced mutations in C_101_, H_138_, and D_170_ and found that each of these mutations completely abolished the ability of RavJ to induce actin cytoskeleton rearrangements (Figure 1). These results indicate that ectopic expression of RavJ triggers the formation of actin punctas in the cell cortex by a mechanism that requires its C_101_H_138_D_170_ motif.

### *ravJ* is dispensable for intracellular growth of *L. pneumophila*

**Figure 2:**
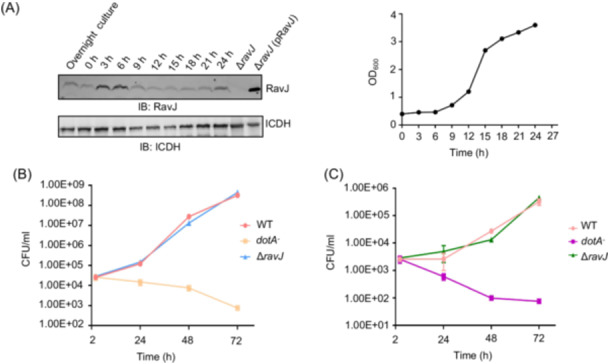
*ravJ* is dispensable for optimal intracellular growth of *Legionella pneumophila*. (A) Growth of *L. pneumophila* LP02 in AYE broth (right) and the expression of RavJ in broth‐grown bacteria (left). Stationary phase cultures were diluted 1:20 into a fresh medium, and bacterial growth was monitored by measuring OD_600_ at the indicated time points. (B, C) ∆*ravJ* mutant displaying no growth defect compared to the wild‐type strain in standard host cells. Bone marrow‐derived macrophages (BMDMs) (B) or *Dictyostelium discoideum* (C) was infected with the indicated *L. pneumophila* strains. Lp03 is an isogenic *dotA* ^‐^ mutant of Lp02. At each time point, host cells were lysed with 0.02% saponin for 30 min, and intracellular bacterial loads were quantified by plating serial dilutions for colony‐forming units (CFUs). Error bars represent SEM; *n* = 3.

To examine the role of *ravJ* in *L. pneumophila* virulence, we first determined the level of RavJ at different growth phases throughout its growth cycle in a bacterial medium. RavJ was detectable in all growth phases (OD_600_ of 0.5–3.5) but became highly expressed in the lag phase (OD_600_ of 0.5–0.7) (Figure 2A) after saturated cultures were diluted into a fresh medium, suggesting that RavJ functions in the initial phase of infection.

We also determined the role of *ravJ* in *L. pneumophila* virulence by examining intracellular bacterial replication of relevant bacterial strains. The Lp02∆*ravJ* mutant exhibited growth comparable to the wild‐type strain (Figure 2B,C), suggesting that, like many Dot/Icm effectors, RavJ is not essential for efficient intracellular replication in standard tissue culture hosts.

### Ectopically expressed RavJ crosslinks with actin

**Figure 3:**
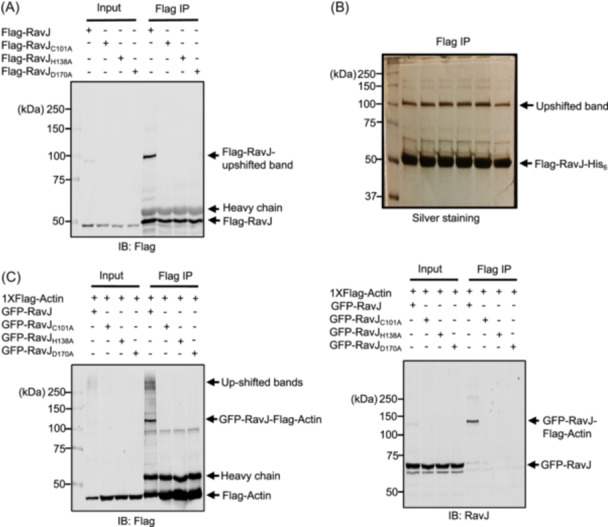
RavJ induces a molecular weight shift in actin by a putative TG activity. (A) Portion of RavJ migrated as a higher molecular weight forms when expressed in mammalian cells. HEK293T cells were transfected to express Flag‐RavJ, RavJ_C101A_, RavJ_H138A_, or the RavJ_D170A_ mutants. Total cell lysates were immunoprecipitated with beads coated with an anti‐Flag antibody. Products were resolved by SDS‐PAGE and probed with an anti‐Flag antibody. Note that RavJ displayed as an upshifted band on the blot. (B) Tandem purification of the Flag‐RavJ‐His_6_ upshifted band. HEK293T cells were transfected with Flag‐RavJ‐His_6_, and cell lysates were immunoprecipitated using Flag antibody‐conjugated beads. Proteins were then eluted from the beads with 3XFLAG peptides and incubated with Ni^2+^‐NTA beads. Products were separated by SDS‐PAGE and detected by silver staining. (C) RavJ forms a covalent complex with actin in mammalian cells. HEK293T cells expressing the indicated proteins were immunoprecipitated using Flag antibody‐conjugated beads and analyzed by SDS‐PAGE. Samples were probed with the anti‐Flag (left) and anti‐RavJ (right) antibody.

TGs function to catalyze homo‐ or heterologous protein crosslink by a transglutamination reaction^26^. The key to understanding the mechanism of how RavJ induces actin rearrangement is to identify its target proteins. To achieve this goal, we expressed Flag‐tagged RavJ or the RavJ_C101A_, RavJ_H138A_, and RavJ_D170A_ mutants in HEK293T cells. Cell lysates were subjected to immunoprecipitation (IP) using anti‐Flag antibody‐conjugated beads. Detection of the precipitated proteins by immunoblotting with the Flag antibody revealed that a portion of wild‐type RavJ but not the mutant migrated as higher molecular weight (MW) forms (Figure 3A), suggesting it is posttranslationally modified. To identify the modification associated with the upshifted protein, we expressed double‐tagged Flag‐RavJ‐His_6_ in HEK293T cells and used a two‐step sequential purification procedure to obtain upshifted RavJ (Figure 3B). Mass spectrometry (MS) analysis of proteins in the upshifted band identified abundant actin and RavJ, suggesting that the upshifted band is a product generated by protein conjugation between these two proteins.

**Figure 4:**
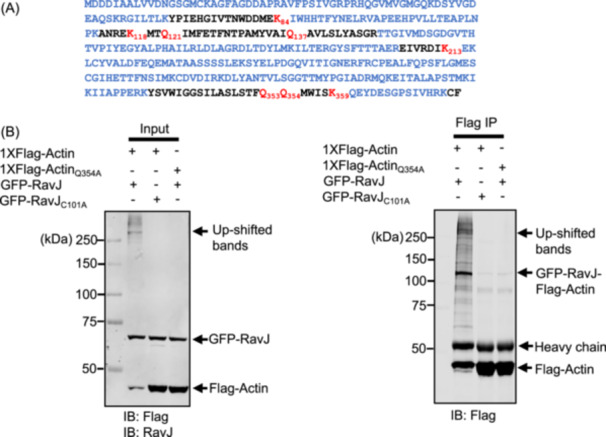
Actin is crosslinked to RavJ through its Gln_354_ residue. (A) Mass spectrometry (MS) analysis of the Flag‐RavJ‐His_6_ upshifted band. The coverage of the amino acids in actin is highlighted in blue. Note that Lys_84_, Lys_118_, Lys_213_, Lys_359_, Gln_121_, Gln_137_, Gln_353_, and Gln_354_ were not recovered from the MS, indicating them as potential linkage residues. (B) Actin–RavJ complex formation assessed by immunoblotting. Notably, the actin Gln354Ala mutant showed a marked reduction in modification (lane 3).

To test whether the upshifted band is a crosslinked product from actin and RavJ, we coexpressed Flag‐tagged actin and GFP fusion of RavJ or its mutants in HEK293T cells. Cell lysates were subjected to IP using agarose beads coated with the Flag‐specific antibody. We found that GFP‐RavJ indeed was linked with actin (Figure 3C). Interestingly, we also observed some up‐shifted bands above 250 kDa, suggesting that RavJ catalyzes the crosslink of actin with multiple proteins. To firstly identify the chemical linkage between actin and RavJ, the protein band corresponding to the RavJ upshifted band was excised, digested with trypsin, and analyzed by MS. TGs mediate protein crosslinking by catalyzing the formation of an isopeptide bond bridge between a lysine (Lys) donor residue on one protein and a glutamine (Gln) acceptor residue on another^26^. Thus, we hypothesized that a lysine residue and a glutamine residue are involved in the crosslink between RavJ and actin. In the MS analysis, around 80% of the peptides in actin were recovered from the tryptic digestion, indicating that Lys_84_, Lys_118_, Lys_213_, Lys_359_, Gln_121_, Gln_137_, Gln_353_, or Gln_354_ in actin is either the potential donor lysine residue or the acceptor glutamine residue (Figure 4A). We then created a series of actin mutants in which each of these sites was replaced with alanine and tested their ability to form the crosslink products. These efforts identified Gln_354_ as the residue critical for forming the conjugate with RavJ (Figures 4B and S1A,B).

### RavJ induces crosslink between actin and members of the Motin family protein

**Figure 5:**
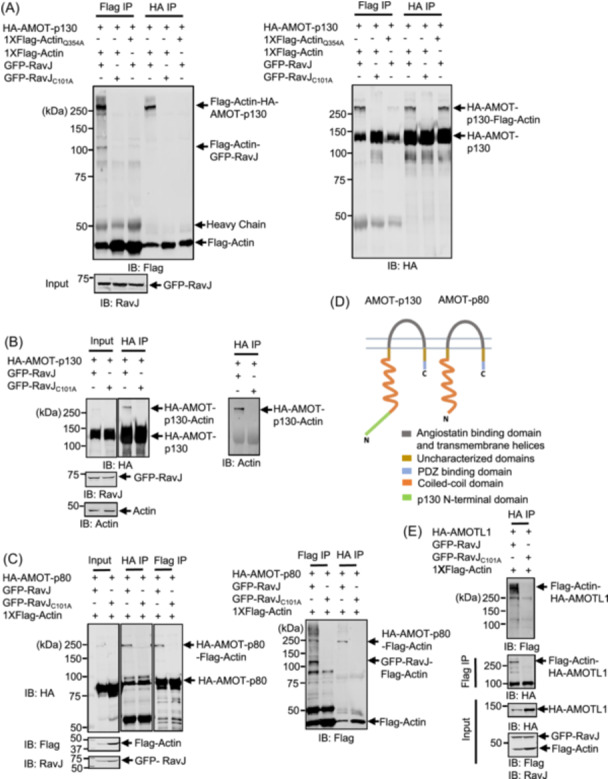
RavJ catalyzes crosslinks between actin and members of the Motin protein family. (A) Actin conjugates with AMOT‐p130 induced by RavJ. Lysates from HEK293T cells co‐transfected with the indicated constructs were immunoprecipitated using either Flag or HA antibody‐conjugated beads. Samples were separated by SDS‐PAGE and analyzed by immunoblotting with anti‐Flag or anti‐HA antibodies. (B) RavJ induces crosslinks between HA‐AMOT and endogenous actin. Cells expressing HA‐AMOT‐p130 and GFP‐RavJ or the GFP‐RavJ_C101A_ mutant were lysed and immunoprecipitated with beads coated with the HA antibody. Crosslinked products were probed by immunoblotting using either anti‐Actin or anti‐Flag antibodies. (C) Actin forms a conjugate with AMOT‐p80 in HEK293T cells in the presence of RavJ. Cells transfected to express the indicated proteins were lysed and subjected to immunoprecipitation (IP) with beads coated with anti‐Flag or anti‐HA antibodies, followed by immunoblotting analysis. The proteins were detected using antibodies specific to Flag, RavJ, or HA. (D) Schematic view of the two isoforms of AMOT produced by alternative splicing of the *AMOT* mRNA. The two isoforms, AMOT‐p130 and AMOT‐p80, contain a conserved coiled‐coil domain, a C‐terminal PDZ binding domain, and an angiostatin binding domain. The AMOT‐p130 isoform harbors a unique N‐terminal domain. (E) RavJ catalyzes a crosslink between AMOTL1 and actin. Cells transfected to express the indicated proteins were lysed and subjected to reciprocal IP, followed by SDS‐PAGE analysis, and were probed with the indicated antibodies. AMOT, Angiomotin; AMOTL1, Angiomotin‐like 1.

TGs normally catalyze crosslinks between two proteins, and in the absence of the receptor substrate, they induce crosslinks between itself and the available donor substrate^30^. Our analysis of the precipitated products obtained by RavJ identified a number of proteins (Figure 3C), one or more of which could be the second substrate that crosslinks with actin in the reaction induced by RavJ. To identify such proteins, we purified the crosslink products using a tandem purification method from HEK293T cells transfected to express Flag‐HA‐Actin and GFP‐RavJ. Samples similarly transfected to express the catalytically inactive RavJ_C101A_ mutant were used as controls. Twenty‐four hours after transfection, cell lysates were subjected to IP with beads coated with an anti‐Flag antibody. Proteins eluted with the 3XFLAG peptide were further purified by IP using an anti‐HA antibody. Samples separated by SDS‐PAGE were detected by immunoblotting with the appropriate antibodies. In samples transfected to express GFP‐RavJ, several upshifted bands were detected with the anti‐Flag antibody (Figure S2A). The gels containing upshifted proteins were excised, digested with trypsin, and analyzed by MS, which allowed us to obtain a list of proteins that potentially crosslink with actin (Table 1). Two components of the Wiskott–Aldrich syndrome and SCAR homolog (WASH) complex, WASHC4 and WASHC5, and one protein from the Motin family, AMOT, were among the proteins identified with high confidence. We considered these proteins as potential substrates of RavJ because of their relevance to the actin cytoskeleton network. Further analysis showed that RavJ did not detectably induce crosslink between actin and WASHC4 or WASHC5 (Figure S2B,C). Importantly, we found that AMOT was able to form a conjugate with actin when coexpressed with RavJ (Figure 5A), suggesting that AMOT is the second substrate of the crosslinking reaction catalyzed by RavJ.

**Table 1:** Top hits from MS analysis of tandem purification.

Protein name	Ratio (RavJ/RavJ_C__101A_)	MS/MS count RavJ_C__101A_	MS/MS count RavJ	Molecular weight (kDa)
Actin	29.68	25	742	42
Splicing factor, proline‐ and glutamine‐rich	19.5	2	39	76
Kinesin‐like protein KIF11	17.5	6	105	119
Adenylyl cyclase‐associated protein 1	7.78	14	109	51
Cytoplasmic dynein 1 heavy chain 1	∞	0	155	532
Eukaryotic translation initiation factor 4 gamma 1	∞	0	84	158
Filamin‐A	∞	0	63	280
**WASH complex subunit strumpellin (WASHC5)**	∞	0	52	134.28
Talin‐1	∞	0	44	270
Insulin receptor substrate 4	∞	0	42	133
**WASH complex subunit 7 (WASHC4)**	∞	0	35	136.4
Afadin	∞	0	22	210
Eukaryotic translation initiation factor 4 gamma 2	∞	0	19	98
General transcription factor II‐I	∞	0	18	108
Vigilin	∞	0	17	141
Src substrate cortactin	∞	0	16	57
**Angiomotin**	∞	0	5	130

Bold text in first column are proteins known to be associated with the cytoskeleton. MS, mass spectrometry; WASH, Wiskott‐Aldrich syndrome and SCAR homolog.

To verify that AMOT is targeted by RavJ, we tested the crosslink between endogenous actin and AMOT. In cells transfected to express GFP‐RavJ, crosslink products formed by HA‐AMOT and endogenous actin were detected (Figure 5B). We also observed a crosslink between actin and AMOT‐p80 (Figure 5C), an AMOT isoform that arises from the alternative splicing of the *AMOT* transcript (Figure 5D). In addition, we tested AMOTL1, which is another member in the Motin family having significant homology with AMOT. Crosslink products of AMOTL1 and actin were detected in HEK293T cells expressing wild‐type RavJ but not its catalytically inactive mutants (Figure 5E). Taken together, our results indicate that RavJ catalyzes the crosslink between actin and members of the Motin family.

**Figure 6:**
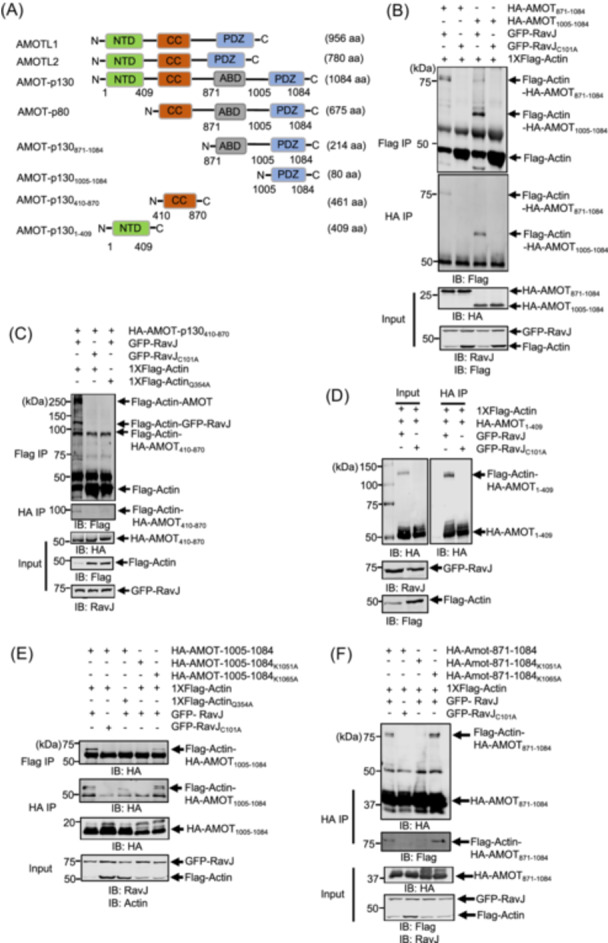
Identification of AMOT modification sites. HEK293T cells were transfected with the indicated constructs, and cell lysates were subjected to reciprocal co‐IP using Flag or HA‐conjugated beads. Crosslinked products were detected by immunoblotting with the specified antibodies. (A) Domain architecture of the Motin family of proteins and the AMOT‐p130 truncations. The HA‐tagged truncation mutants were made as indicated. (B) PDZ binding domain is enough to crosslink with actin. (C) AMOT coiled‐coil truncation (AMOT_410‐870_) crosslinks with actin in the presence of RavJ. (D) RavJ catalyzes the formation of a covalent bond between the N‐terminal domain of AMOT‐p130 and actin. (E) Lys1051Ala mutation in the PDZ binding domain loses its ability to crosslink with actin. (F) Lys1051Ala mutation in the AMOT_871‐1084_ truncation abolishes its ability to crosslink with actin.

The Motin family of proteins harbor several structural domains, including the N‐terminus domain potentially involved in Yes‐associated protein 1 (YAP1) binding^31^, a conserved coiled‐coil (CC) domain, and the C‐terminal PDZ‐binding domain^32^ (Figure 6A). To determine the crosslink site on AMOT, we constructed a number of HA‐tagged AMOT truncation mutants (Figure 6A) and tested their ability to crosslink with actin in cells expressing RavJ. Our results showed that all four truncations were able to crosslink with actin (Figure 6B–D). To identify the modification sites on each truncation, the corresponding crosslink products were subjected to MS analysis (Figure S3A–C). However, despite multiple attempts using different enzymes to digest the crosslink products, we were unable to determine the crosslink sites between the two proteins. The mass of the peptide without the crosslink is too big to extract the peptide from the gel and sequence it. We then mutated the two lysine residues in the truncation that contains the PDZ‐binding domain (Figure 6E). Substitution of Lys_1051_ abolished the production of the crosslinked product (Figure 6E). The same mutation in the AMOT_871‐1084_ truncation also abolished the crosslink (Figure 6F), suggesting that AMOT_871‐1084_ crosslinks with actin through its Lys_1051_ residue.

### RavJ catalyzes the crosslink between purified actin and AMOT

**Figure 7:**
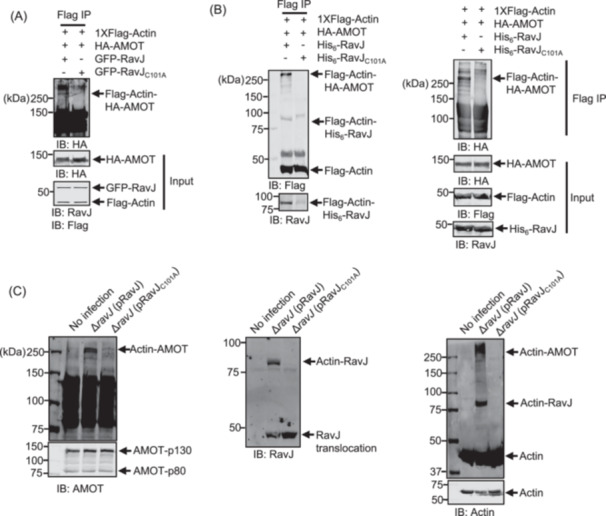
RavJ induces the formation of a protein conjugate between actin and AMOT. (A) RavJ catalyzes the crosslink between actin and AMOT in a cell‐free system. Cells transfected to express Flag‐Actin, HA‐AMOT, GFP‐RavJ, or GFP‐RavJ_C101A_ were lysed with RIPA buffer without EDTA. As indicated, cell lysates were combined and incubated at 37°C for 2 h. Samples were then immunoprecipitated using Flag antibody‐conjugated beads and analyzed by immunoblotting with the specified antibodies. (B) RavJ induces a crosslink between purified actin and AMOT in vitro. Reactions containing Flag‐Actin, HA‐AMOT, His_6_‐RavJ, or His_6_‐RavJ_C101A_ were incubated at 37°C for 2 h. Samples were immunoprecipitated with beads coated with an anti‐Flag antibody. The crosslink products were detected by the indicated antibodies. (C) Endogenous actin‐AMOT crosslinking occurs in cells infected with *L. pneumophila* overexpressing RavJ. HEK293T cells expressing the FcγII receptor were infected with the indicated bacterial strains at an MOI of 50 for 4 h. Following lysis with 0.2% saponin, samples were analyzed by SDS‐PAGE and immunoblotting using the indicated antibodies. MOI, multiplicity of infection.

We next examined whether the crosslink between actin and AMOT occurs in cell‐free reactions. HEK293T cells transfected to express Flag‐Actin, HA‐AMOT, GFP‐RavJ, or GFP‐RavJ_C101A_ were lysed, and the lysates were mixed and incubated at 37°C for 2 h. Beads coated with the Flag antibody were used to enrich Flag‐Actin, and crosslinking products formed by actin and AMOT were detected by immunoblotting. The protein conjugate detectable by the HA‐specific antibody was only present in reactions that received lysates containing wild‐type RavJ (Figure 7A), indicating that the RavJ‐induced crosslink between actin and AMOT occurs in a cell‐free system.

To further determine the activity of this TG, we established reactions in which recombinant RavJ purified from *Escherichia coli* was incubated with Flag‐Actin and HA‐AMOT purified from HEK293T cells, and the production of a crosslink product was detected after the reactions were allowed to proceed for 2 h at 37°C. Consistent with the results from earlier experiments with cell lysates, adding His_6_‐RavJ but not His_6_‐RavJ_C101A_ to the reactions caused a crosslink between these two proteins (Figure 7B). Together, these results establish that RavJ is a TG that catalyzes a crosslink between AMOT and actin.

### *L. pneumophila* induces crosslinking between actin and AMOT in a RavJ‐dependent manner

Our results from ectopic expression by transfection strongly suggest that RavJ catalyzes a protein crosslink between AMOT and actin. To determine whether this reaction occurs under physiological conditions, we examined RavJ activity during *L. pneumophila* infection. HEK293T cells expressing the FcγII receptor were infected with opsonized bacteria from the indicated *L. pneumophila* strains at a multiplicity of infection (MOI) of 50. No crosslink between AMOT and actin was detected in cells infected with strain Lp03, an avirulent *dotA* mutant^33^, or the wild‐type strain Lp02^34^ (Figure S4). Considering the possibility that the amount of crosslinked products was too low to be detected in samples infected with strain Lp02, we examined whether overexpression of RavJ in the wild‐type strain allows us to detect the crosslink products. Indeed, infection of the cells with the strain Lp02∆*ravJ* (pRavJ) led to a robust crosslink between AMOT and actin (Figure 7C). In contrast, a crosslink did not occur in cells similarly infected with the strain Lp02∆*ravJ* (pRavJ_C101A_), which overexpressed the enzymatically inactive RavJ mutant (Figure 7C). These results indicate that RavJ catalyzes a crosslink between actin and AMOT in cells infected with *L. pneumophila* competent in the Dot/Icm system that overexpressed RavJ. The amount of crosslink products in cells infected with the wild‐type strain likely was not sufficient for detection with our method.

### Simultaneous knockdown of *AMOT* and *AMOTL1* interferes with the formation of actin patches induced by RavJ

**Figure 8:**
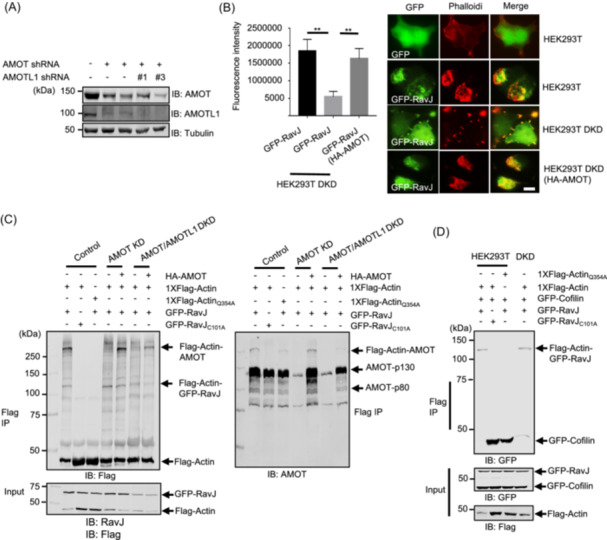
Knockdown of AMOT and AMOTL1 reduces actin accumulation in HEK293T cells induced by RavJ. (A) Expression level of AMOT and AMOTL1 examined by Western blot. Protein expression was examined in cell lysates from two clones of AMOT knockdown (KD) and AMOT/AMOTL1 double knockdown (DKD) cells. Proteins were detected by an AMOT‐p130‐specific antibody and an AMOTL1‐specific antibody. Tubulin was detected as a loading control. (B) AMOT/AMOTL1 DKD in HEK293T cells significantly reduces actin accumulation in cells expressing wild‐type RavJ. Cells transfected to express the indicated proteins were fixed and stained with phalloidin conjugated with Texas red (the right panel). Bar, 10 µm. The phalloidin fluorescence signal was quantified by Image J software to evaluate the amount of F‐actin (the left panel). Data are the mean ± SEM (**p < 0.01). (C) AMOT/AMOTL1 DKD in HEK293T cells abolishes the crosslink between actin and the Motin family of proteins. Cells were transfected to express the indicated proteins and samples were subjected to IP with beads coated with an anti‐Flag antibody. Crosslinking products were detected with the Flag‐specific antibody and AMOT‐specific antibodies. (D) RavJ inhibits the binding between actin and cofilin. Cells transfected to express the indicated proteins were lysed and subjected to IP with beads coated with the Flag‐specific antibody. The binding between actin and cofilin was detected by the indicated antibodies.

Ectopic expression of RavJ in mammalian cells led to the formation of actin patches (Figure 1). The observation that RavJ induces a crosslink between AMOT and actin suggests that this event is important for the actin polymerization phenotype. To examine the relevance between the protein crosslink and the formation of actin patches, we determined the impact of AMOT depletion on RavJ‐induced actin patch formation. To this end, we introduced shRNAs that target mRNAs of both AMOT and AMOTL1 into HEK293T cells, which are known to express high levels of these two isoforms but almost an undetectable level of AMOTL2^35^. Introduction of shRNAs by lentiviral transduction led to a significant reduction of the protein levels of both AMOT and AMOTL1 (Figure 8A). Next, we transfected cells with GFP‐RavJ and assessed actin patch formation using phalloidin staining. In cells that received AMOT and AMOTL1 shRNAs, actin patch formation was dramatically reduced (Figure 8B). Importantly, the crosslink between actin and AMOT became undetectable in cells receiving shRNAs targeting AMOT (Figure 8C). Furthermore, overexpression of AMOT by transfection rescued not only the RavJ‐induced formation of actin patches but also the crosslink between actin and AMOT (Figure 8B,C). Taken together, these results support the conclusion that members of the Motin family of proteins are important for RavJ‐induced actin accumulation by forming protein conjugates with actin.

### RavJ interferes with the interaction between actin and cofilin

Earlier studies suggest that actin interacts with profilin and cofilin through its Gln_354_ residue^36^, ^37^, ^38^. Cofilin depolymerizes and severs actin filaments while profilin binds to actin monomers and provides ATP‐actin for incorporation into actin filaments^39^. The fact that actin crosslinks with RavJ and AMOT via Gln_354_ inspired us to investigate whether RavJ affects the binding between actin and actin‐binding proteins. HA‐profilin‐1, HA‐profilin‐2, or GFP‐cofilin was expressed in HEK293T cells along with the relevant proteins, and cell lysates were subjected to IP using an anti‐Flag antibody. Interestingly, expression of RavJ reduced the binding between actin and cofilin (Figure 8D), but not profilin‐1 and profilin‐2 (Figure S5A,B), suggesting that RavJ‐induced actin accumulation in the cell cortex was a result of reduced actin turnover.

### LegL1 blocks the activity of RavJ by sterically hindering its catalytic site

**Figure 9:**
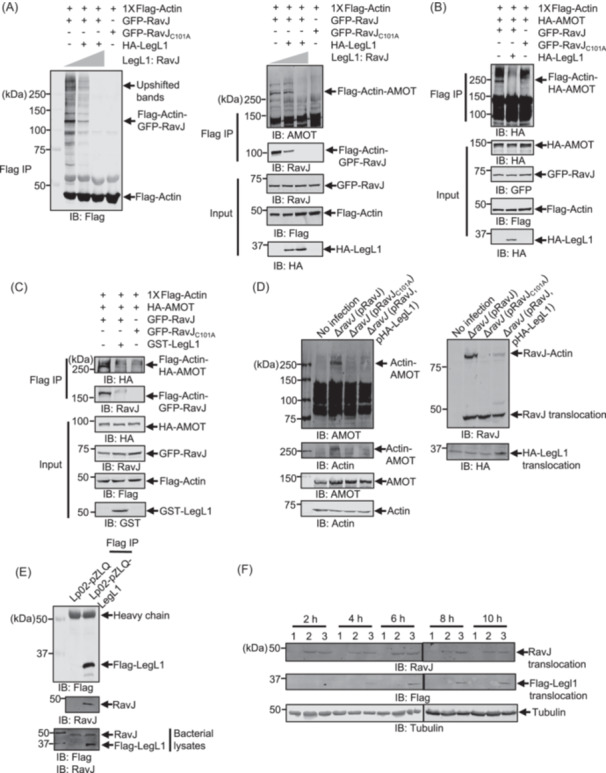
LegL1 antagonizes the catalytic activity of RavJ. (A) Expression of LegL1 in HEK293T cells reduce crosslink products induced by RavJ. Cell lysates expressing the indicated proteins were immunoprecipitated using beads conjugated with the anti‐Flag antibody, separated by SDS‐PAGE, and analyzed using the indicated antibodies. (B) LegL1 inhibits the activity of RavJ in a cell‐free system. Cells transfected to express Flag‐Actin, HA‐LegL1, HA‐AMOT, GFP‐RavJ, or GFP‐RavJ_C101A_ were lysed with RIPA buffer without EDTA. Lysates of HA‐LegL1 were pre‐incubated with those expressing GFP‐RavJ for 1 h at 37°C. Reactions containing the indicated cell lysates were immunoprecipitated with beads coated with the Flag antibody and proteins were detected with the indicated antibodies. (C) Overexpression of LegL1 in *L. pneumophila* inhibits the enzymatic activity of RavJ. Indicated *L. pneumophila* strains were opsonized and used to infect HEK293T cells expressing the FcγII receptor at an MOI of 50 for 4 h. Cells lysed with 0.2% saponin were probed with the indicated antibodies. (D) Recombinant LegL1 reduces the crosslink products induced by RavJ. Cells transfected to express Flag‐Actin, HA‐AMOT, GFP‐RavJ, or GFP‐RavJC101A were lysed with RIPA buffer without EDTA. 10 μg of GST‐LegL1 was pre‐incubated with cell lysates of GFP‐RavJ for 1 h at 37°C. Reactions containing the indicated components were immunoprecipitated using beads coated with an anti‐Flag antibody and proteins were detected with the indicated antibodies. (E) LegL1 directly binds to RavJ in *L. pneumophila*. Bacterial cells carrying either the vector pZLQ or pZLQ‐LegL1 were lysed and then immunoprecipitated with beads coated with the Flag antibody. Binding between RavJ and LegL1 was detected by a RavJ‐specific antibody and an anti‐Flag antibody, respectively. (F) Overexpression of LegL1 in *L. pneumophila* does not influence the translocation of RavJ. HEK293T cells transfected to express the FcγII receptor were treated with different bacterial strains: 1, no infection; 2, wild‐type *L. pneumophila*; 3, wild type *L. pneumophila* overexpressing Flag‐LegL1. Cells were collected at the indicated time points and lysed by 0.2% saponin. RavJ and LegL1 translocations were detected by RavJ‐specific antibodies and the Flag‐specific antibody, respectively. Tubulin was probed as a loading control.

In a previous study, LegL1 (Lpg0945) was identified as a putative metaeffector of RavJ, which interacts with its amino terminus containing the predicted motif important for its enzymatic activity^29^. We then tested whether LegL1 can influence actin cytoskeleton rearrangement induced by RavJ. Coexpression of HA‐LegL1 with GFP‐RavJ significantly reduced the formation of crosslink products by actin and AMOTs and such reduction became more apparent as the ratio between LegL1 and RavJ increased (Figure 9A), indicating that LegL1 inhibits the activity of RavJ. The inhibitory effect of LegL1 can be attributed to at least two potential mechanisms: LegL1 reverses the crosslink by an enzymatic activity or it inhibits the activity of RavJ by direct binding.

To distinguish between these two possibilities, we pre‐incubated cell lysates expressing HA‐LegL1 with cell lysates expressing GFP‐RavJ for 1 h at 37°C. Cell lysates expressing HA‐AMOT and Flag‐Actin were then added to the cell‐free system. RavJ, which has been pre‐incubated with HA‐LegL1, was unable to induce a crosslink between actin and AMOT, suggesting that LegL1 blocks the activity of RavJ (Figure 9B). Furthermore, the addition of HA‐LegL1 to reactions containing crosslinked actin‐AMOT did not detectably reduce the amount of the conjugate product (Figure S6). We also tested the activity of recombinant LegL1 purified from *E. coli*. Incubation of GST‐LegL1 with cell lysates expressing GFP‐RavJ ablated its ability to catalyze the crosslink between actin and AMOT (Figure 9C).

We next examined the interactions between LegL1 and RavJ in *L. pneumophila*. RavJ can be coimmunoprecipitated (co‐IP) from lysates of *L. pneumophila* expressing Flag‐LegL1 with beads coated with an anti‐Flag antibody, indicating binding between LegL1 and RavJ (Figure 9E). To determine whether LegL1 blocks RavJ's TG activity during *L. pneumophila* infection, we examined their interaction in host cells. HEK293T cells expressing the FcγII receptor were infected with opsonized wild‐type *L. pneumophila* or wild‐type *L. pneumophila* overexpressing Flag‐LegL1 at an MOI of 50. Four hours after infection, cell lysates were subjected to IP with beads coated with the Flag antibody. We found that HA‐LegL1 indeed interacts with RavJ in infected cells (Figure S7A). Consistent with this observation, GFP‐RavJ and HA‐LegL1 co‐expressed in HEK293T also interact as assayed by Co‐IP (Figure S7B). We further tested the effect of LegL1 on the RavJ‐induced crosslink during bacterial infection. HEK293T cells were infected with the *ravJ* mutant complemented with RavJ, the RavJ_C101A_ mutant, or both RavJ and LegL1, at an MOI of 50. The crosslink between AMOT and actin was only detected in cells infected with the ∆*ravJ* (pRavJ) strain but not the strain ∆*ravJ* (pRavJ, pLegL1) (Figure 9D), suggesting that LegL1 effectively inhibits the crosslink caused by RavJ during bacterial infection. Finally, we examined whether LegL1 affects the delivery of RavJ into host cells by *L. pneumophila*. Overexpression of LegL1 did not detectably impact the translocation of RavJ into host cells (Figure 9F). Taken together, these results indicate that LegL1 inhibits the activity of RavJ by direct binding, and such inhibition likely only occurs after protein translocation has occurred.

## DISCUSSION

The actin cytoskeleton network is a major host structural component that provides structural and functional support in numerous vital cellular activities. It also directs the trafficking of cargo‐containing vesicle trafficking throughout the cell by functioning as a cellular highway^40^. Intracellular bacterial pathogens have evolved remarkable strategies to subvert the host cytoskeletal machinery to promote bacterial internalization, facilitate the biogenesis of bacteria‐containing phagosomes, and co‐opt actin‐dependent movement to benefit pathogen dissemination^41^. To support diverse infection events, intracellular pathogens hijack the actin cytoskeleton by introducing effector proteins into the host cytosol by specialized secretion systems^9^. Among these, *Coxiella burnetii* triggers actin reorganization at the attachment site of human macrophages by binding to CR3 receptors to stimulate bacterial internalization^42^. After entering host cells, this bacterium replicates within an acidic compartment called the parasitophorous vacuole (PV) in these immune cells. Optimal intracellular growth of *C. burnetii* requires F‐actin accumulation around the PV, but the detailed mechanisms are not fully characterized^43^. *Chlamydia* species replicate in a host membrane‐derived compartment termed inclusion. Optimal development and maintenance of vacuole morphology and integrity require F‐actin rings surrounding the inclusion to stabilize the organelle^44^, ^45^. Spotted Fever Group *Rickettsia*, such as *R. rickettsii* and *R. conorii*, escape the phagosome before lysosomal fusion after entering a host cell. After the escape, these bacteria induce actin polymerization to form an actin tail that facilitates bacterial motility within the cell^46^, ^47^.

Actin is organized in two primary forms, the monomeric G‐actin and the filamentous form F‐actin^48^. Polymerization and depolymerization of actin filaments are kept in a dynamic balance to tightly regulate movement and other cell functions^49^. The VCA domains of N‐WASP (Wiskott–Aldrich syndrome protein) and SCAR/WAVE (suppressor of the cAMP receptor/WASP‐family verprolin homologous protein) activate the Arp2/3 complex, promoting the formation of new F‐actin branches from existing mother filaments^50^. Actin‐depolymerizing factor ADF/cofilin regulates actin dynamics by depolymerizing filaments at their pointed ends, thereby restoring a pool of actin monomers for filament assembly^40^. The actin molecule consists of two main lobes divided by a prominent upper cleft, with each lobe further subdivided into four distinct subdomains, designated SD1 through SD4^50^. SD1, which is built from residues 1–32, 70–144, and 338–375, forms an important target area for a large number of actin‐binding proteins such as profilin, cofilin, and gelsolin^50^. Of note is that the RavJ‐induced actin crosslink with AMOT occurs at Gln_354_, a site located on SD1, suggesting that this modification may disrupt interactions between actin subunits in filaments and actin‐binding proteins. Supporting this idea, cofilin binding to actin is markedly diminished (Figure 8D), indicating that RavJ functions to block actin depolymerization, resulting in the accumulation of F‐actin in the cell cortex.

It is well‐established that *L. pneumophila* avoids the delivery of its vacuole to lysosomes by modulating the ER‐to‐Golgi vesicle trafficking^51^. Shortly after being internalized by a host cell, the plasma membrane‐derived vacuole containing *L. pneumophila* is converted into a compartment that has a similarity to the ER–Golgi intermediate compartment^52^, ^53^, ^54^. The biogenesis of this specialized phagosome has been studied extensively^54^. A repertoire of Dot/Icm effectors has been shown to directly hijack the host vesicle trafficking pathway^2^, ^55^, and some of them affect this cellular process indirectly to bypass the microbicidal endosomal compartment^56^. The modulation of the actin cytoskeleton clearly contributes to the development of the LCV. For example, the metal protease RavK cleaves actin, abolishing its ability to form actin polymers^12^, whereas LegK2 phosphorylates components of the actin nucleator ARP2/3 complex and thus inhibits actin polymerization on the phagosome^13^. Ceg14 inhibits actin polymerization by depleting ATP in the reaction via a mechanism catalyzed by its S‐HxxxE motif^14^, ^57^. These proteins are very likely working synergistically to temporally inhibit actin polymerization on the LCV and thus preventing fusion with late endosomes^13^. In contrast to the inhibitory effects of RavK and LegK2, VipA enhances actin polymerization by acting as an actin nucleator^15^. RavJ also appears to promote the formation of actin filaments. Here, we show that the RavJ‐induced crosslink between actin and AMOT blocks the depolymerization activity of ADF/cofilin, resulting in the stabilization of actin polymers in the cell cortex (Figure S8).

Although induction of actin punctas by ectopically expressed RavJ allowed us to investigate its biochemical activity, we were unable to detect this phenotype in cells infected by *L. pneumophila*. The inability to detect accumulation of actin punctas under infection conditions likely is due to a low level of RavJ injected into infected cells or such accumulation is too subtle to be detected. Nevertheless, the observation that the knockdown of the AMOT family abolished the RavJ‐induced formation of actin puncta validated the importance of the crosslink between AMOT and actin in this phenotype. It is known that *L. pneumophila* regulates the actin cytoskeleton structure with effectors with diverse and sometimes opposite activity^7^, ^12^, ^13^, ^15^, ^16^, and the lack of a detectable phenotype induced by RavJ likely is the net outcome of the activities of these effectors.

Balanced modulation of host processes has recently emerged as a prominent feature associated with the interactions between *L. pneumophila* and its hosts. In some cases, the balance is achieved by effector pairs with opposite biochemical activity, which may function at different phases of infection^58^. In other cases, the importance of balance lies in the cellular locations of the molecular events regulated by the effectors^19^. In addition to other effectors that inhibit actin polymerization, the regulation imposed by RavJ is controlled by its metaeffector LegL1, which blocks its function by direct protein–protein interactions. LegL1 likely functions to spatially and temporally prevent RavJ from inducing excessive actin polymerization.

## MATERIALS AND METHODS

### Media, bacteria strains, and cell lines

For molecular cloning, *E. coli* strain DH5a was used, while XL1‐Blue and BL21(DE3) strains were employed for recombinant protein expression and purification. *E. coli* cultures were grown in LB broth or on LB agar, supplemented with antibiotics when required (ampicillin at 100 μg/ml; kanamycin at 30 μg/ml). All *L. pneumophila* strains used in this study were derived from the Philadelphia 1 strain Lp02^34^. The *L. pneumophila* strain Lp03, an isogenic *dotA* ^−^ mutant of Lp02^33^, was used alongside other derivatives of Lp02. Bacteria were cultured on CYE plates or in ACES‐buffered yeast extract (AYE) broth, as previously described^34^. Kanamycin (30 μg/ml) was used for selection, and thymidine (100 μg/ml) was added when required for thymidine‐auxotrophic strains. The *ravJ* in‐frame deletion mutant was generated using a two‐step allelic exchange method, as described previously^59^. HEK293T cells (ATCC) were maintained in Dulbecco's modified minimal Eagle's medium (DMEM) supplemented with 10% fetal bovine serum (FBS). Bone marrow‐derived macrophages (BMDMs) were isolated and cultured, as described previously^60^. All cell lines were routinely tested for mycoplasma contamination using a commercial mycoplasma detection kit (ATCC, cat# 30‐1012 K).

### Plasmid constructions

A comprehensive list of plasmids, bacterial strains, and antibodies used in this study is provided in Tables S1 and S2. For protein purifications, *ravJ*, *ravJ*_*C101A*_, and *legl1* were cloned into pQE30 (QIAGEN) or pGEX6p‐1. For complementation assays, *ravJ* and the C101A mutant were cloned into pZLQ‐Flag, a modified version of pZLQ^61^ containing a Flag tag^62^. The *legl1* coding sequence was cloned into either pZLQ‐Flag or pZL507^63^ for overexpression in *L. pneumophila*. For ectopic protein expression in mammalian cells, genes were cloned into various vectors, including 4XFlag CMV^22^, pFlag‐CMV (Sigma), pEGFP‐C1 (Clontech) vector, 3XHA pCDNA3.1 vector^64^, or pAPH^65^, a derivative of pVR1012 suitable for expressing proteins with an amino‐terminal HA tag. Human *AMOT* and *AMOTL1* were amplified from cDNAs of HEK293T cells and then inserted into *Bam*HI/*Sal*I of pAPH. For the shRNA knockdown of *AMOTL1* in mammalian cells, the pLKO.1‐hygro vector (Addgene, plasmid #24150) was used to generate the pLKO.1‐hygro‐*AMOTL1*‐sh construct. Packing plasmids psPAX2 (Addgene, plasmid #12260) and pMD2.G (Addgene, plasmid #12259) were used for lentiviral construct transduction.

### Transfection, IP, infection

HEK293T cells grown to about 90% confluence were transfected with different plasmids using Lipofectamine 3000 (Thermo Fisher Scientific). Transfected cells were harvested 18–24 h post‐transfection and lysed in RIPA buffer (Thermo Fisher Scientific). For IP, lysates were incubated overnight at 4°C with agarose beads conjugated to anti‐Flag (Sigma‐Aldrich, cat# F2426) or anti‐HA (Pierce, cat# 88836) antibodies, as needed. Beads were washed three times with ice‐cold RIPA buffer. For tandem purification, followed by RIPA wash, agarose beads with Flag‐tagged proteins were washed with Flag‐to‐His buffer (100 mM Na‐Phosphate, pH 8.0, 150 mM NaCl, 0.05% Triton X‐100) three times and eluted with 3XFLAG peptides (Sigma‐Aldrich, cat# F3290). The elution fraction was then subjected to HA beads or Ni^2+^‐NTA agarose beads (QIAGEN) as needed. Beads were resolved by SDS‐PAGE, followed by immunoblotting with specific antibodies or silver staining according to the manufacturer's instructions (Sigma‐Aldrich, cat# PROTSIL1).

For infection assays, *L. pneumophila* strains were cultured in AYE broth to a post‐exponential phase (OD_600_ of 3.2–3.8). Complemented and overexpressed strains were induced with 0.5 mM IPTG for 2 h at 37°C before infection. HEK293T cells were transfected to express the FcγII receptor^22^, and *L. pneumophila* strains were opsonized with *L. pneumophila*‐specific antiserum (1:500) for 30 min at 37°C. Infections were carried out at an MOI of 50 for 4 h, after which cells were lysed with 0.2% saponin. Lysates were resolved by SDS‐PAGE and analyzed by immunoblotting.

### Antibodies and immunoblotting

Rabbit polyclonal antibodies against purified His_6_‐RavJ were generated by Pocono Rabbit Farm & Laboratory using standard immunization protocols. The antibodies were affinity‐purified, as described before^66^. For immunoblotting, SDS‐PAGE‐resolved samples were transferred to 0.2 μm nitrocellulose membranes (Bio‐Rad, cat# 1620112). Membranes were blocked in 5% nonfat milk and probed with the following primary antibodies: anti‐HA (Sigma‐Aldrich, cat# H3663, 1:5000), anti‐Flag (Sigma‐Aldrich, cat# F1804, 1:5000), anti‐RavJ (prepared in this study, 1:5000), anti‐AMOTL1 (Sigma‐Aldrich, cat# SAB1408393, 1:5000), anti‐AMOT (Abnova, cat# H00154796‐B01P, 1:5000), anti‐tubulin (DSHB, clone E7, 1:10,000), anti‐actin (MP Biomedicals, cat# 0869100, 1:5000), anti‐ICDH (1:10,000)^63^, anti‐GFP (1:10,000)^63^, and anti‐GST^63^. IRDye‐conjugated secondary antibodies were used, and signals were visualized using the Odyssey infrared imaging system (Li‐COR Biosciences).

### Immunostaining

HEK293T cells were plated at 1 × 10^5^ per well on glass coverslips in 24‐well plates and transfected with the indicated constructs for 24 h. Cells were rinsed three times with PBS, fixed in 4% formaldehyde for 30 min at room temperature, and then permeabilized with 0.3% Triton X‐100 for 15 min. Following three additional PBS washes, cells were blocked in 5% goat serum for 1 h. F‐actin was stained with Texas Red‐conjugated phalloidin (Thermo Fisher Scientific, cat#T7471) at 1:500 for 1 h at room temperature. Images were captured using an Olympus X‐81 fluorescence microscope.

### Protein purification

Overnight *E. coli* cultures (10 ml) were inoculated into 400 ml of LB medium containing either 100 μg/ml ampicillin or 30 μg/ml kanamycin and grown to an OD_600nm_ of 0.8–1.0. Protein expression was induced with 0.5 mM IPTG, followed by incubation at 18°C for 16–18 h. Cells were harvested by centrifugation at 12,000*g*, lysed by sonication, and the lysates were clarified by two rounds of centrifugation at 12,000*g* for 20 min at 4°C. His_6_‐tagged proteins were purified by incubating the supernatant with Ni^2+^‐NTA beads for 2 h at 4°C, followed by washes with TBS containing 20 mM imidazole and elution with 300 mM imidazole in TBS. Eluted proteins were dialyzed overnight at 4°C in TBS supplemented with 5% glycerol and 1 mM DTT. GST‐tagged proteins were purified using glutathione beads (Pierce, cat# 16101) for 2 h at 4°C and eluted with buffer containing 50 mM Tris (pH 8.0), 0.4 M NaCl, 50 mM reduced glutathione, 0.1% Triton X‐100, and 1 mM DTT.

### shRNA knockdown of *AMOT* and *AMOTL1*

MISSION shRNA retroviral constructs targeting *AMOT* were purchased from Sigma‐Aldrich (Clone ID: NM_133265.1‐1628s1c1). To collect the viral supernatant, cells were seeded at 2 × 10^5^ cells/6 cm plate (around 10% confluency). After overnight culture, cells were transfected with the retroviral construct targeting *AMOT* along with two packing plasmids, psPAX2 and pMD2.G. The viral supernatant was collected at Day 3 and 4 after transfection and was filtered with a 0.45‐μm syringe filter. The titer of the produced lentivirus was determined by using a Lenti‐X Gostix Plus Titer Kit (Takara, cat# 631281). To generate *AMOT* knockdown cell lines, HEK293T cells were seeded at 2 × 10^5^ cells/10 cm plate (around 10% confluency). Media was removed on Day 2, and viral supernatant was added to cover the whole plate (3.5 ml/10 cm plate). Viral supernatant was added every 3 h for three times. On Day 3, media were replaced with fresh media. Cells were selected with media supplemented with puromycin (InvivoGen, cat# ant‐pr‐1) for a few days, and single colonies were selected.

To generate *AMOTL1* knockdown cell lines, the pLKO.1‐hygro‐*AMOTL1*‐sh construct was generated by cloning annealed oligos 5′‐CCGGTCCGGGCCCATCCTACAAACAACTTTCTCGAGAAAGTTGTTTGTAGGATGGGCTTTTTTGG‐3′ and 5′‐AATTCCAAAAAAGCCCATCCTACAAACAACTTTCTCGAGAAAGTTGTTTGTAGGATGGGCCCGGA‐3′ into the pLKO.1‐hygro vector. Viral supernatant was generated by transfecting HEK293T cells with the retroviral construct targeting *AMOTL1* along with the packing plasmids, psPAX2 and pMD2.G. The viral supernatant was collected, as described above. The *AMOT* knockdown cell line was infected with the viral supernatant as described above, and single colonies were selected with media supplemented with hygromycin (Thermo Fisher Scientific, cat#10687010).

### Cell‐free assays and in vitro assays

In the cell‐free assays, HEK293T cells expressing Flag‐Actin, HA‐AMOT, GFP‐RavJ, GPF‐RavJ_C101A_, and HA‐LegL1 were lysed by RIPA buffer without EDTA. Cell lysates were spun down at 12,000*g*, and the supernatants were collected. Reactions with combined supernatants as indicated were allowed to proceed for 2 h at 37°C. The supernatants were then subjected to IP using anti‐Flag or anti‐HA antibody‐coated beads, as needed.

For in vitro assays, HEK293T cells expressing HA‐AMOT or Flag‐Actin were lysed in RIPA buffer and subjected to HA‐IP or Flag‐IP, as needed. Proteins were eluted from the corresponding beads using 3XFLAG peptides or HA peptides (Thermo Fisher Scientific). 5 μg of His_6_‐RavJ or His_6_‐RavJ_C101A_ was added to the in vitro assay, and the reaction was left in the 37°C incubator for 2 h. The in vitro reaction was then subjected to Flag‐IP followed by SDS‐PAGE analysis.

### Intracellular bacterial growth assay

*L. pneumophila* strains were grown to the post‐exponential phase (OD_600_ of 3.2–3.8) before infection. BMDMs isolated from female A/J mice as described before^60^ were seeded onto 24‐well plates and were infected with relevant *L. pneumophila* strains at an MOI of 0.05 at 37°C. Cells were collected at the indicated time points and lysed with 0.02% saponin for half an hour on ice. The number of bacteria was determined by counting the colony‐forming unit (CFU) of appropriately diluted saponin‐soluble fractions.

### LC‐MS/MS analysis

Protein bands were subjected to in‐gel trypsin digestion for protein identification. Peptides were reconstituted in a solution of 96.9% water, 3% acetonitrile (ACN), and 0.1% formic acid (FA) at a final concentration of 0.2 μg/μl. A total of 1 μg of peptides was analyzed using a Dionex UltiMate 3000 RSLC nano system coupled to a Q Exactive™ HF Hybrid Quadrupole‐Orbitrap mass spectrometer (Thermo Scientific), as previously described^67^, ^68^. Peptides were first loaded onto a trap column (300 μm ID × 5 mm, 5 μm, 100 Å PepMap C18) and then separated on a 50 cm × 75 µm analytical column (2 µm 100 Å PepMap C18) maintained at 50°C.

The mobile phases were solvent A (0.1% FA in water) and solvent B (0.1% FA in 80% ACN). Peptides were loaded using a buffer of 98% water, 2% ACN, and 0.1% FA at 5 μl/min for 5 min. Elution was performed at 150 nl/min with a 130‐min gradient: 5.1%–27% solvent B over 80 min, 27%–45% over 20 min, 45%–100% over 5 min, held at 100% B for 7 min, then returned to 2% solvent B at 112 min, and held for 18 min for re‐equilibration. The mass spectrometer was operated in a positive ion mode with data‐dependent acquisition (DDA) and Advanced Peak Detection enabled, targeting the top 20 precursors. Fragmentation was performed using a stepped normalized collision energy of 27%. Orbitrap resolution was set to 120,000 for MS1 and 15,000 for MS2. MS1 scans were acquired from 350 to 1600 m/z, with an isolation window of 1.2 m/z and a fixed first mass of 100 m/z for MS2. Spray voltage was set at 2 kV, with Automatic Gain Control (AGC) targets of 4e5 (MS1) and 5e4 (MS2).

For protein identification, raw data were processed using MaxQuant^69^ (v1.6.3.3) against the *Homo sapiens* database from UniProt. Searching parameters included a 10 ppm precursor mass tolerance, trypsin specificity with up to two missed cleavages, carbamidomethylation of cysteine (fixed), and methionine oxidation (variable). The false discovery rate (FDR) was set at 1% for both peptide spectral matches (PSMs) and proteins. Quantification was based on unique and razor peptides, and label‐free quantification (LFQ) intensity values were used to estimate relative protein abundance. Only proteins identified with at least one unique peptide and MS/MS spectral count ≥2 were considered for downstream analysis.

## AUTHOR CONTRIBUTIONS

**Yan Liu**: Conceptualization; data curation; formal analysis; investigation; methodology; writing—original draft; writing—review and editing. **Yao Liu**: Conceptualization; data curation; methodology; writing—review and editing. **Zhao‐Qing Luo**: Conceptualization; funding acquisition; project administration; supervision; writing—original draft; writing—review and editing.

## ETHICS STATEMENT

No animal or human research was involved in this study.

## CONFLICT OF INTERESTS

The authors declare no conflict of interests.

## Supporting information

Table S1‐plasmids‐Yan.

Table S2 Bacterial strains and antibodies‐Yan.

**Figure S2.** Identification of the cellular targets of RavJ. (A) Tandem purification of actin crosslink products catalyzed by RavJ. HEK293T cells co‐transfected to express Flag‐HA‐Actin, GFP‐RavJ, or GFP‐RavJ_C101A_ were subjected to immunoprecipitation using beads conjugated with an anti‐Flag antibody. Proteins were eluted from the beads by the 3XFLAG peptide. Elution fractions were then immunoprecipitated with beads coated with the HA‐specific antibody. Products resolved by SDS‐PAGE were detected by silver staining (right) or probed with the Flag antibody (left). The gel plug corresponding to the upshifted band was cut for MS analysis. (B, C) WASHC4 and WASHC5 do not crosslink with actin in the presence of RavJ. HEK293T cells co‐transfected to express the indicated proteins were lysed and immunoprecipitated by beads coated with the Flag‐specific or HA‐specific antibody. Note that there are no crosslink products detected between actin and WASHC4 or WASHC5.
**Figure S3.** Determination of the crosslink sites in AMOT. (A–C) Tandem purification of crosslink products of actin and AMOT truncations. HEK293T cells co‐expressing the indicated proteins were immunoprecipitated using beads conjugated with an anti‐Flag antibody. Proteins were eluted from the beads by the 3XFLAG peptide. Elution fractions were then immunoprecipitated with beads coated with the HA‐specific antibody. Products resolved by SDS‐PAGE were detected by silver staining (right) or probed with the indicated antibodies (left). Protein bands corresponding to the crosslink products were excised for MS analysis.
**Figure S4.** Determination of the effect of RavJ during bacterial infection. HEK293T cells transfected to express the FcγII receptor were treated with opsonized bacterial strains as indicated. Cells were collected at 4 h after infection and were lysed by 0.2% saponin. RavJ and SidC translocations were detected by RavJ‐specific or SidC‐specific antibodies. Tubulin was probed as a loading control.
**Figure S5.** Effects of RavJ on the binding between actin and actin‐binding proteins. (A, B) RavJ does not influence the binding between actin and actin‐binding proteins, profilin 1 and profilin 2. HEK293T cells co‐transfected to express the indicated proteins were lysed and subjected to IP with beads coated with a Flag‐specific antibody.
**Figure S6.** LegL1 does not reverse the protein crosslink induced by RavJ. HEK293T cell lysates from cells expressing Flag‐Actin and GFP‐RavJ (or GFP‐RavJ_C101A_) were mixed with lysates from cells expressing HA‐LegL1. After a 2‐h incubation, the lysates were subjected to IP with beads coated with the Flag‐specific antibody. Note that the expression of HA‐LegL1 did not reverse the pre‐existing crosslink between AMOT and actin.
**Figure S7.** LegL1 blocks the transglutaminase activity of RavJ post‐translocation. (A) HEK293T cells transfected to express the FcγII receptor were treated with opsonized bacterial strains as indicated. Cells were collected at 4 h after infection and were lysed by 0.2% saponin. RavJ and Flag‐LegL1 translocation were detected by RavJ‐specific or Flag‐specific antibodies. Actin was probed as a loading control. (B) HEK293T cells transfected to express the indicated proteins were lysed and subjected to IP with beads coated with the HA‐specific antibody. Binding between HA‐LegL1 and GFP‐RavJ was detected by the indicated antibodies.
**Figure S8.** Model of RavJ in actin cytoskeleton modulation. RavJ catalyzes the crosslink between actin and AMOT, which led to the blockage of the ADF/cofilin binding site in actin, resulting in the stabilization of actin fibers in the cell cortex.

**Figure S1.** Determination of the crosslink site in actin. (A, B) HEK293T cells co‐transfected to express the indicated proteins were lysed and immunoprecipitated by beads coated with the Flag‐specific antibody. Note that only the Gln354Ala mutation in actin abolished the crosslink products induced by RavJ.

## Data Availability

All the data are available in the main text and Supporting Information. All unique constructs and cell lines described in this article are available upon reasonable request from academic researchers. Please contact the corresponding author at luoz@purdue.edu for request of any materials.
