# Supplementary material for: Legionella pneumophila modulates the host cytoskeleton by an effector of transglutaminase activity
Source: mLife. 2025 Jun 18;4(3):232–48. doi: 10.1002/mlf2.70013 (PMC12207909; doi:10.1002/mlf2.70013)
Supplement: Supplementary file 1 — Table S1‐plasmids‐Yan. [file MLF2-4-232-s001.doc]

| TableS1 Plasmids used in this study | | |
| --- | --- | --- |
|
| Plasmid | Relevant phenotypes | Sources |
| pSR47s | *ori*R6K, *ori*T RP4, Kan^R^, *SacB* | [1] |
| pQE30 | For expression of His_6_-tagged protein in *E. coli* | Qiagen |
| pJB908 | Amp, *thy*^+^ | [2] |
| pZLQ-Flag | For expression of Flag-tagged protein in *L. pneumophila* | [3] |
| pZL507 | For expression of His_6_-tagged protein in *L. pneumophila* | [4] |
| pEGFPC-1 | Mammalian expression vector with an N-terminal GFP tag | Clontech |
| pAPH | Mammalian expression vector with N-terminal HA tag | [5] |
| pCMV-Flag | Mammalian expression vector with N-terminal Flag tag | Sigma |
| pCMV-4XFlag | Mammalian expression vector with N-terminal Flag tag | [6] |
| pGEX-6p-1 | For expression of GST-tagged protein in *E. coli* | [4] |
| psPAX2 | Lentiviral packaging vector | Addgene |
| pMD2.G | Lentiviral packaging vector | Addgene |
| pLKO.1-hygro | Lentiviral vector | Addgene |
| 3XHA pCDNA3.1 | Mammalian expression vector with N-terminal HA tag | [7] |
| pZLΔ*ravJ* | Construct used for in-frame deletion of *ravJ* | This study |
| pZL2201 | pLKO.1-puro-*AMOT*-sh | Sigma |
| pZL2202 | pLKO.1-hygro-*AMOTL1*-sh | This study |
| pZL2203 | pQE30-RavJ | This study |
| pZL2204 | pQE30-RavJ_C101A_ | This study |
| pZL2205 | pGEX-6p-1-LegL1 | This study |
| pZL2206 | pZLQ-Flag-RavJ | This study |
| pZL2207 | pZLQ-Flag-RavJ_C101A_ | This study |
| pZL2208 | pZLQ-Flag-LegL1 | This study |
| pZL2209 | pZL507-His_6_-LegL1 | This study |
| pZL2210 | pEGFPC1-RavJ | This study |
| pZL2211 | pEGFPC1-RavJ_C101A_ | This study |
| pZL2212 | pEGFPC1-RavJ_H138A_ | This study |
| pZL2213 | pEGFPC1-RavJ_D170A_ | This study |
| pZL2214 | pEGFPC1-Cofilin | This study |
| pZL2215 | pAPH-AMOT-p130 | This study |
| pZL2216 | pAPH-AMOT-p80 | This study |
| pZL2217 | pAPH-AMOT-p130_1-406_ | This study |
| pZL2218 | pAPH-AMOT-p130_410-870_ | This study |
| pZL2219 | pAPH-AMOT-p130_871-1084_ | This study |
| pZL2220 | pAPH-AMOT-p130_871-1084 K1051A_ | This study |
| pZL2221 | pAPH-AMOT-p130_871-1084 K1065A_ | This study |
| pZL2222 | pAPH-AMOT-p130_1005-1084_ | This study |
| pZL2223 | pAPH-AMOT-p130_1005-1084 K1051A_ | This study |
| pZL2224 | pAPH-AMOT-p130_1005-1084 K1065A_ | This study |
| pZL2205 | pAPH-PFN1 | This study |
| pZL2206 | pAPH-PFN2 | This study |
| pZL2227 | pCMV-4XFlag-RavJ | This study |
| pZL2228 | pCMV-1XFlag-β-actin | [8] |
| pZL2229 | pCMV-1XFlag-β-actin_Q354A_ | This study |
| pZL2230 | pCMV-1XFlag-HA-β-actin | This study |
| pZL2231 | pCMV-1XFlag-β-actin-His_6_ | This study |
| pZL2232 | pCDNA3.1-3XHA-WASHC4 | This study |
| pZL2233 | pCDNA3.1-3XHA-WASHC5 | This study |

**References:**

1. Duménil G, Isberg RR. The Legionella pneumophila IcmR protein exhibits chaperone activity for IcmQ by preventing its participation in high-molecular-weight complexes. Mol Microbiol. 2001;40(5):1113-27. doi: 10.1046/j.1365-2958.2001.02454.x. PubMed PMID: 11401716.

2. Bardill JP, Miller JL, Vogel JP. IcmS-dependent translocation of SdeA into macrophages by the Legionella pneumophila type IV secretion system. Mol Microbiol. 2005;56(1):90-103. doi: 10.1111/j.1365-2958.2005.04539.x. PubMed PMID: 15773981.

3. Luo ZQ, Farrand SK. Signal-dependent DNA binding and functional domains of the quorum-sensing activator TraR as identified by repressor activity. Proc Natl Acad Sci U S A. 1999;96(16):9009-14. doi: 10.1073/pnas.96.16.9009. PubMed PMID: 10430886; PubMed Central PMCID: PMCPMC17723.

4. Xu L, Shen X, Bryan A, Banga S, Swanson MS, Luo ZQ. Inhibition of host vacuolar H+-ATPase activity by a Legionella pneumophila effector. PLoS Pathog. 2010;6(3):e1000822. Epub 20100319. doi: 10.1371/journal.ppat.1000822. PubMed PMID: 20333253; PubMed Central PMCID: PMCPMC2841630.

5. Wang SH, Wang A, Liu PP, Zhang WY, Du J, Xu S, et al. Divergent Pathogenic Properties of Circulating Coxsackievirus A6 Associated with Emerging Hand, Foot, and Mouth Disease. J Virol. 2018;92(11). Epub 20180514. doi: 10.1128/JVI.00303-18. PubMed PMID: 29563294; PubMed Central PMCID: PMCPMC5952127.

6. Qiu J, Sheedlo MJ, Yu K, Tan Y, Nakayasu ES, Das C, et al. Ubiquitination independent of E1 and E2 enzymes by bacterial effectors. Nature. 2016;533(7601):120-4. Epub 20160406. doi: 10.1038/nature17657. PubMed PMID: 27049943; PubMed Central PMCID: PMCPMC4905768.

7. Sheedlo MJ, Qiu J, Tan Y, Paul LN, Luo ZQ, Das C. Structural basis of substrate recognition by a bacterial deubiquitinase important for dynamics of phagosome ubiquitination. Proc Natl Acad Sci U S A. 2015;112(49):15090-5. Epub 20151123. doi: 10.1073/pnas.1514568112. PubMed PMID: 26598703; PubMed Central PMCID: PMCPMC4679006.

8. Liu Y, Zhu W, Tan Y, Nakayasu ES, Staiger CJ, Luo ZQ. A Legionella Effector Disrupts Host Cytoskeletal Structure by Cleaving Actin. PLoS Pathog. 2017;13(1):e1006186. Epub 20170127. doi: 10.1371/journal.ppat.1006186. PubMed PMID: 28129393; PubMed Central PMCID: PMCPMC5298343.
