# Supplementary material for: Legionella pneumophila modulates the host cytoskeleton by an effector of transglutaminase activity
Source: mLife. 2025 Jun 18;4(3):232–48. doi: 10.1002/mlf2.70013 (PMC12207909; doi:10.1002/mlf2.70013)
Supplement: Supplementary file 2 — Table S2 Bacterial strains and antibodies‐Yan. [file MLF2-4-232-s003.docx]

TableS2 Bacterial strains and antibodies used in this study

| Bacterial Strains | Source | Identifier |
| --- | --- | --- |
| *L. pneumophila* LP02 | [1] | N/A |
| *L. pneumophila* LP03 | [2] | N/A |
| LP02 *ravJ* | This study | N/A |
| LP02 *ravJ* (pZL507) | This study | N/A |
| LP02 *ravJ* (pRavJ)  LP02 *ravJ* (pRavJ, pLegL1) | This study  This study | N/A  N/A |
| LP02 *ravJ* (pRavJ_C101A_) | This study | N/A |
| LP02 (pZLQ-Flag) | This study | N/A |
| LP02 (pZLQ-Flag-LegL1) | This study | N/A |
| *E.coli* BL21(DE3) | NEB | CAT#C2527I |
| *E.coli* XL1-Blue | Agilent | CAT#200249 |

| Antibodies | Source | Identifier |
| --- | --- | --- |
| anti-HA | Sigma | cat# H3663 |
| anti-Flag | Sigma | cat# F1804 |
| anti-ICDH | [3] | N/A |
| anti-tubulin | DSHB | E7 |
| anti-RavJ | This study | N/A |
| anti-Actin | MP Biomedicals | cat# 0869100 |
| anti-AMOT | Abnova | cat# H00154796-B01P |
| anti-AMOTL1 | Sigma-Aldrich | cat# SAB1408393 |
| anti-GST | [3] | [3] |

anti-SidC [4] N/A

1. Berger KH, Isberg RR. Two distinct defects in intracellular growth complemented by a single genetic locus in Legionella pneumophila. Mol Microbiol. 1993;7(1):7-19. doi: 10.1111/j.1365-2958.1993.tb01092.x. PubMed PMID: 8382332.

2. Berger KH, Merriam JJ, Isberg RR. Altered intracellular targeting properties associated with mutations in the Legionella pneumophila dotA gene. Mol Microbiol. 1994;14(4):809-22. doi: 10.1111/j.1365-2958.1994.tb01317.x. PubMed PMID: 7891566.

3. Xu L, Shen X, Bryan A, Banga S, Swanson MS, Luo ZQ. Inhibition of host vacuolar H+-ATPase activity by a Legionella pneumophila effector. PLoS Pathog. 2010;6(3):e1000822. Epub 20100319. doi: 10.1371/journal.ppat.1000822. PubMed PMID: 20333253; PubMed Central PMCID: PMCPMC2841630.

4. Luo ZQ, Isberg RR. Multiple substrates of the Legionella pneumophila Dot/Icm system identified by interbacterial protein transfer. Proc Natl Acad Sci U S A. 2004;101(3):841-6. Epub 20040108. doi: 10.1073/pnas.0304916101. PubMed PMID: 14715899; PubMed Central PMCID: PMCPMC321768.
