# Supplementary material for: Legionella pneumophila modulates the host cytoskeleton by an effector of transglutaminase activity
Source: mLife. 2025 Jun 18;4(3):232–48. doi: 10.1002/mlf2.70013 (PMC12207909; doi:10.1002/mlf2.70013)
Supplement: Supplementary file 3 — Figure S2. Identification of the cellular targets of RavJ. (A) Tandem purification of actin crosslink products catalyzed by RavJ. HEK293T cells co‐transfected to express Flag‐HA‐Actin, GFP‐RavJ, or GFP‐RavJC101A were subjected to immunoprecipitation using beads conjugated with an anti‐Flag antibody. Proteins were eluted from the beads by the 3XFLAG peptide. Elution fractions were then immunoprecipitated with beads coated with the HA‐specific antibody. Products resolved by SDS‐PAGE were detected by silver staining (right) or probed with the Flag antibody (left). The gel plug corresponding to the upshifted band was cut for MS analysis. (B, C) WASHC4 and WASHC5 do not crosslink with actin in the presence of RavJ. HEK293T cells co‐transfected to express the indicated proteins were lysed and immunoprecipitated by beads coated with the Flag‐specific or HA‐specific antibody. Note that there are no crosslink products detected between actin and WASHC4 or WASHC5. Figure S3. Determination of the crosslink sites in AMOT. (A–C) Tandem purification of crosslink products of actin and AMOT truncations. HEK293T cells co‐expressing the indicated proteins were immunoprecipitated using beads conjugated with an anti‐Flag antibody. Proteins were eluted from the beads by the 3XFLAG peptide. Elution fractions were then immunoprecipitated with beads coated with the HA‐specific antibody. Products resolved by SDS‐PAGE were detected by silver staining (right) or probed with the indicated antibodies (left). Protein bands corresponding to the crosslink products were excised for MS analysis. Figure S4. Determination of the effect of RavJ during bacterial infection. HEK293T cells transfected to express the FcγII receptor were treated with opsonized bacterial strains as indicated. Cells were collected at 4 h after infection and were lysed by 0.2% saponin. RavJ and SidC translocations were detected by RavJ‐specific or SidC‐specific antibodies. Tubulin was probed as a loading control. Figure S5. Effe [file MLF2-4-232-s002.pdf]

Supplementary Figure 2

(A)

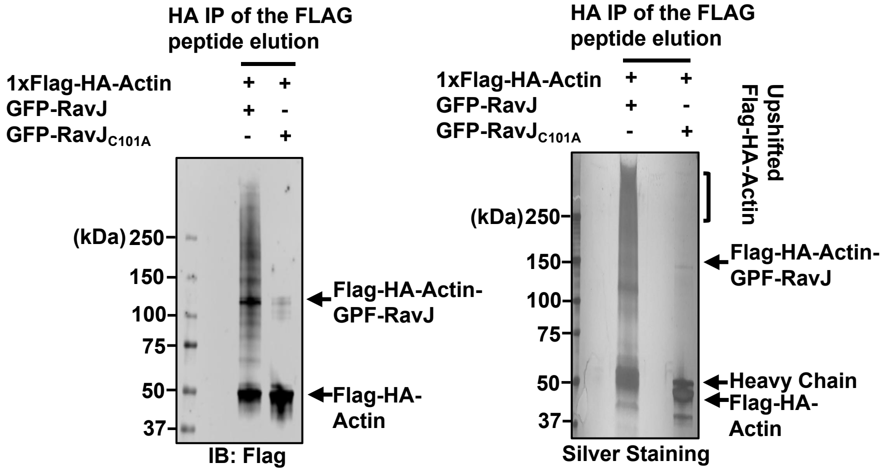

(B)

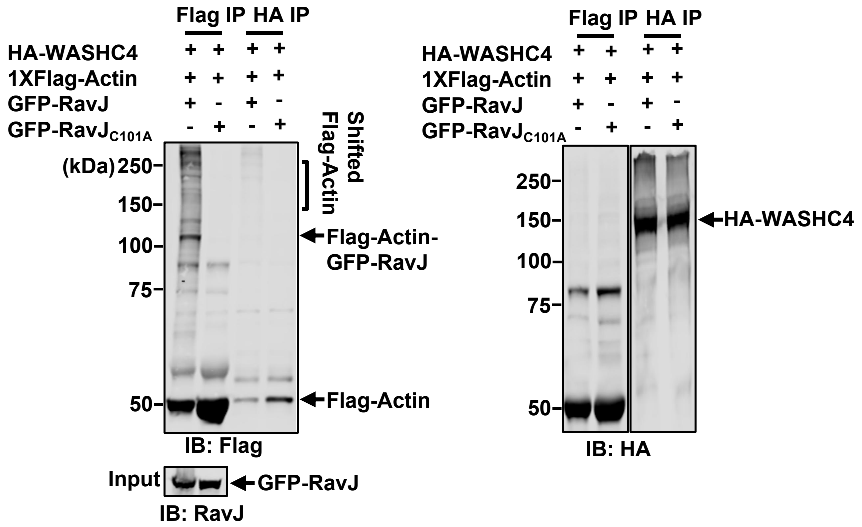

(C)

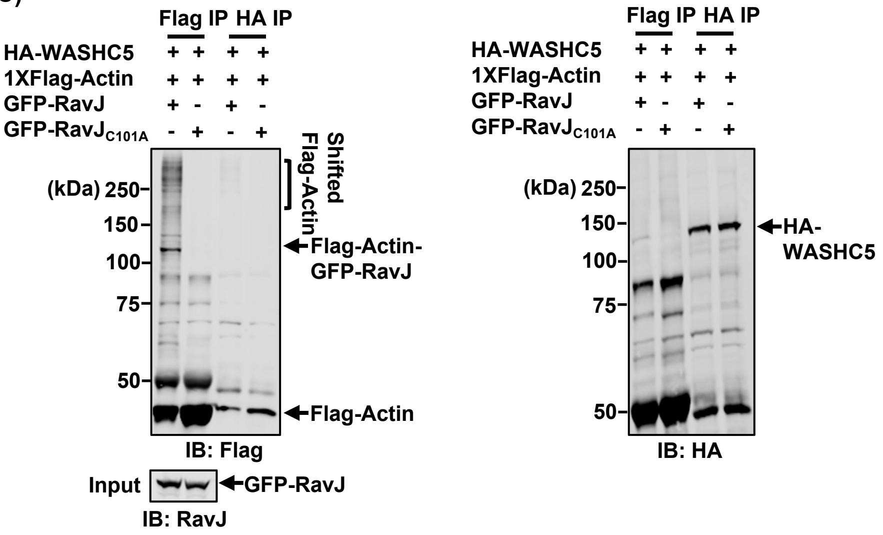

Supplementary Figure 3

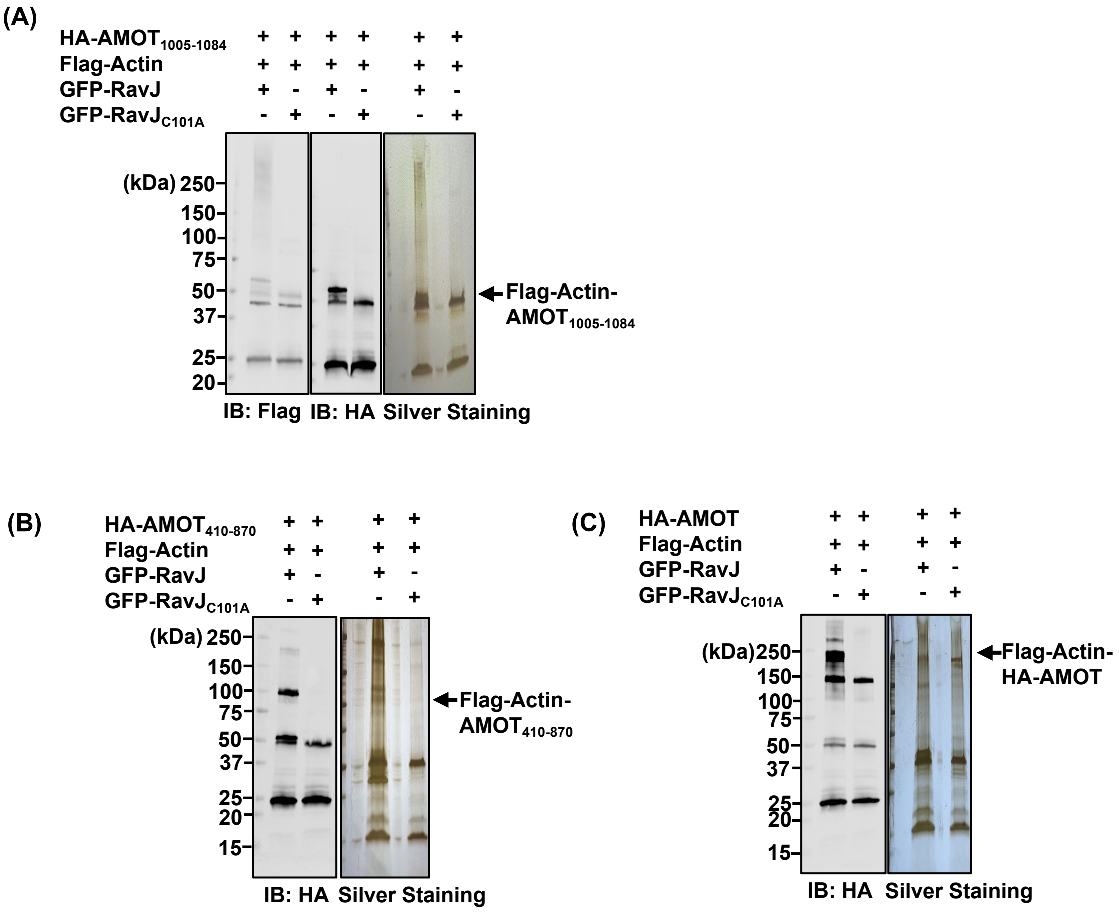

Supplementary Figure 4

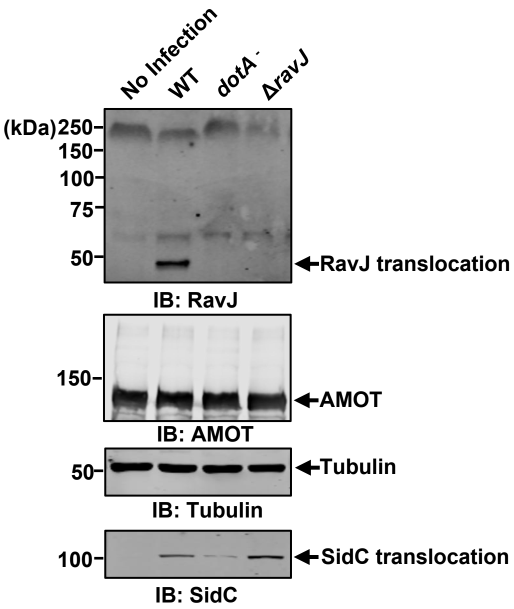

Supplementary Figure 5

(A)

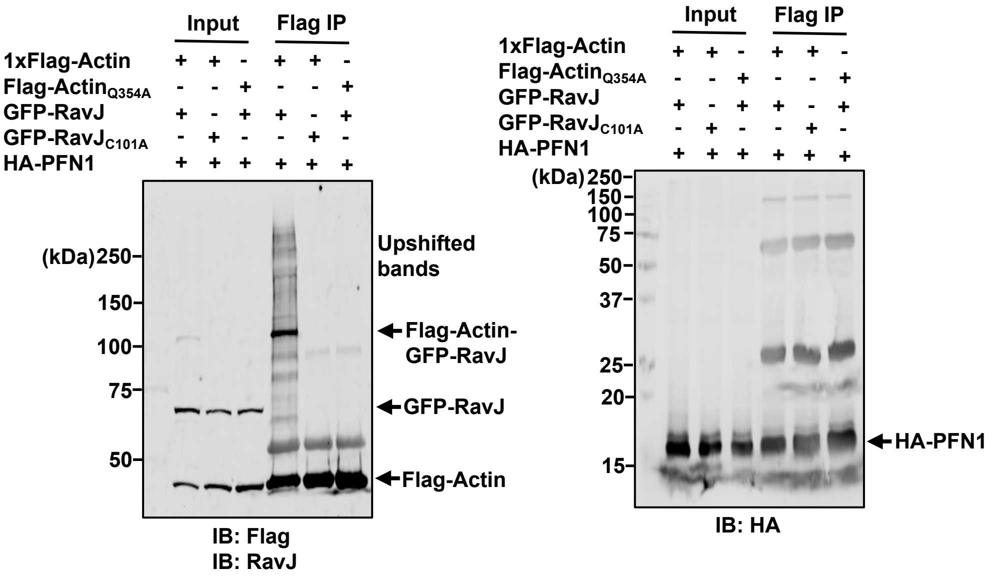

(B)

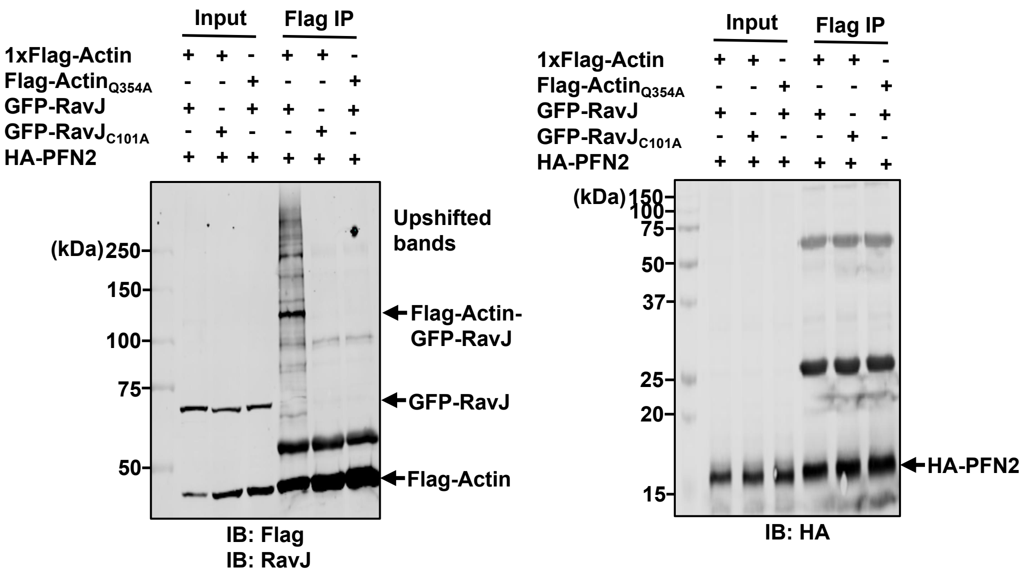

Supplementary Figure 6

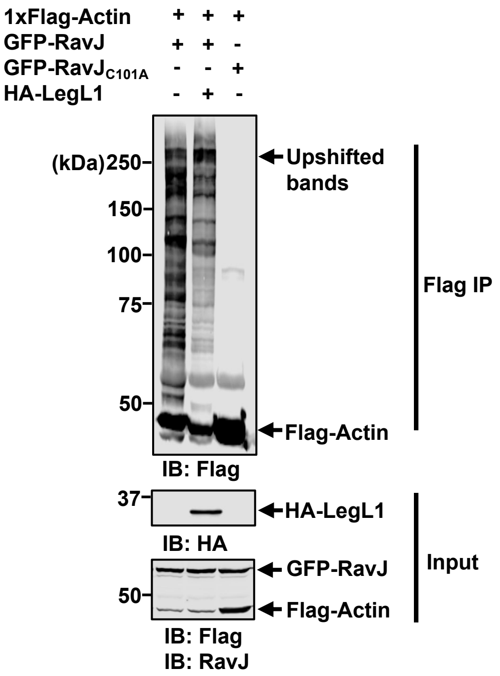

Supplementary Figure 7

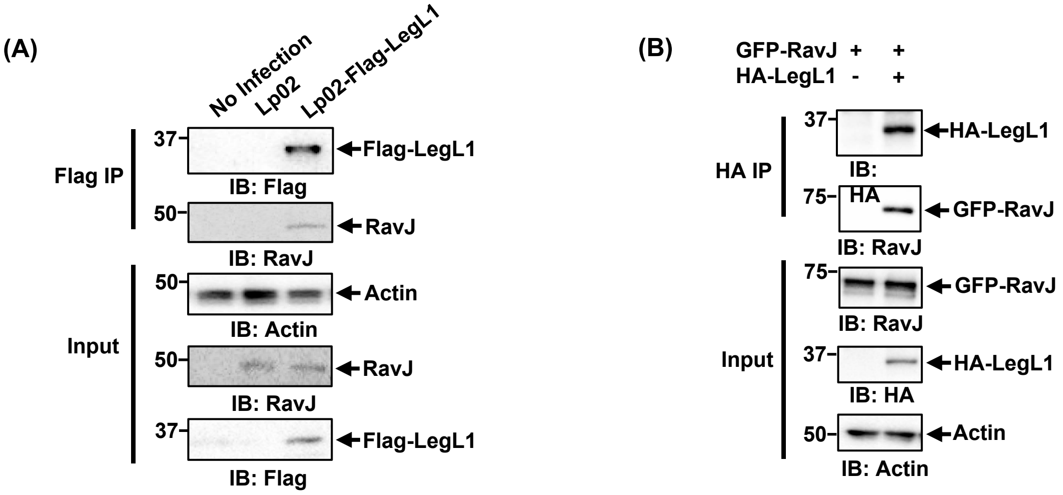

Supplementary Figure 8

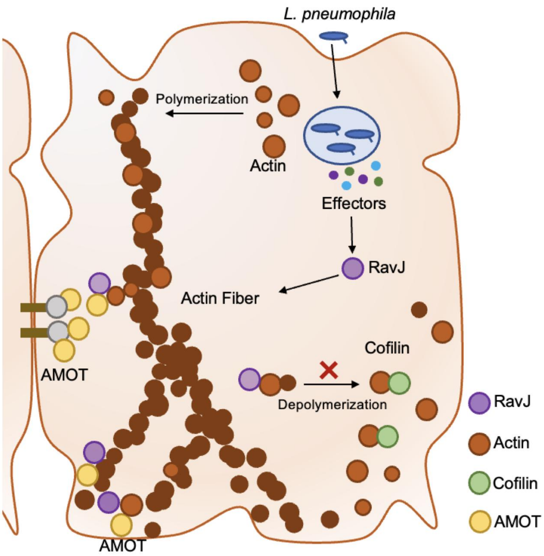

Table 1: Top hits from MS analysis of tandem purification

Protein Names	Ratio (RavJ/$RavJ_{C101A}$)	MS/MS count $RavJ_{C101A}$	MS/MS count RavJ	Mol. weight [kDa]
Actin	29.68	25	742	42
Splicing factor, proline- and glutamine-rich	19.5	2	39	76
Kinesin-like protein KIF11	17.5	6	105	119
Adenylyl cyclase-associated protein 1	7.78	14	109	51
Cytoplasmic dynein 1 heavy chain 1	∞	0	155	532
Eukaryotic translation initiation factor 4 gamma 1	∞	0	84	158
Filamin-A	∞	0	63	280
WASH complex subunit strumpellin (WASHC5)	∞	0	52	134.28
Talin-1	∞	0	44	270
Insulin receptor substrate 4	∞	0	42	133
WASH complex subunit 7 (WASHC4)	∞	0	35	136.4
Afadin	∞	0	22	210
Eukaryotic translation initiation factor 4 gamma 2	∞	0	19	98
General transcription factor II-I	∞	0	18	108
Vigilin	∞	0	17	141
Src substrate cortactin	∞	0	16	57
Angiomotin	∞	0	5	130
