# Supplementary material for: Legionella pneumophila modulates the host cytoskeleton by an effector of transglutaminase activity
Source: mLife. 2025 Jun 18;4(3):232–48. doi: 10.1002/mlf2.70013 (PMC12207909; doi:10.1002/mlf2.70013)
Supplement: Supplementary file 4 — Figure S1. Determination of the crosslink site in actin. (A, B) HEK293T cells co‐transfected to express the indicated proteins were lysed and immunoprecipitated by beads coated with the Flag‐specific antibody. Note that only the Gln354Ala mutation in actin abolished the crosslink products induced by RavJ. [file MLF2-4-232-s004.pptx]

## Slide 1
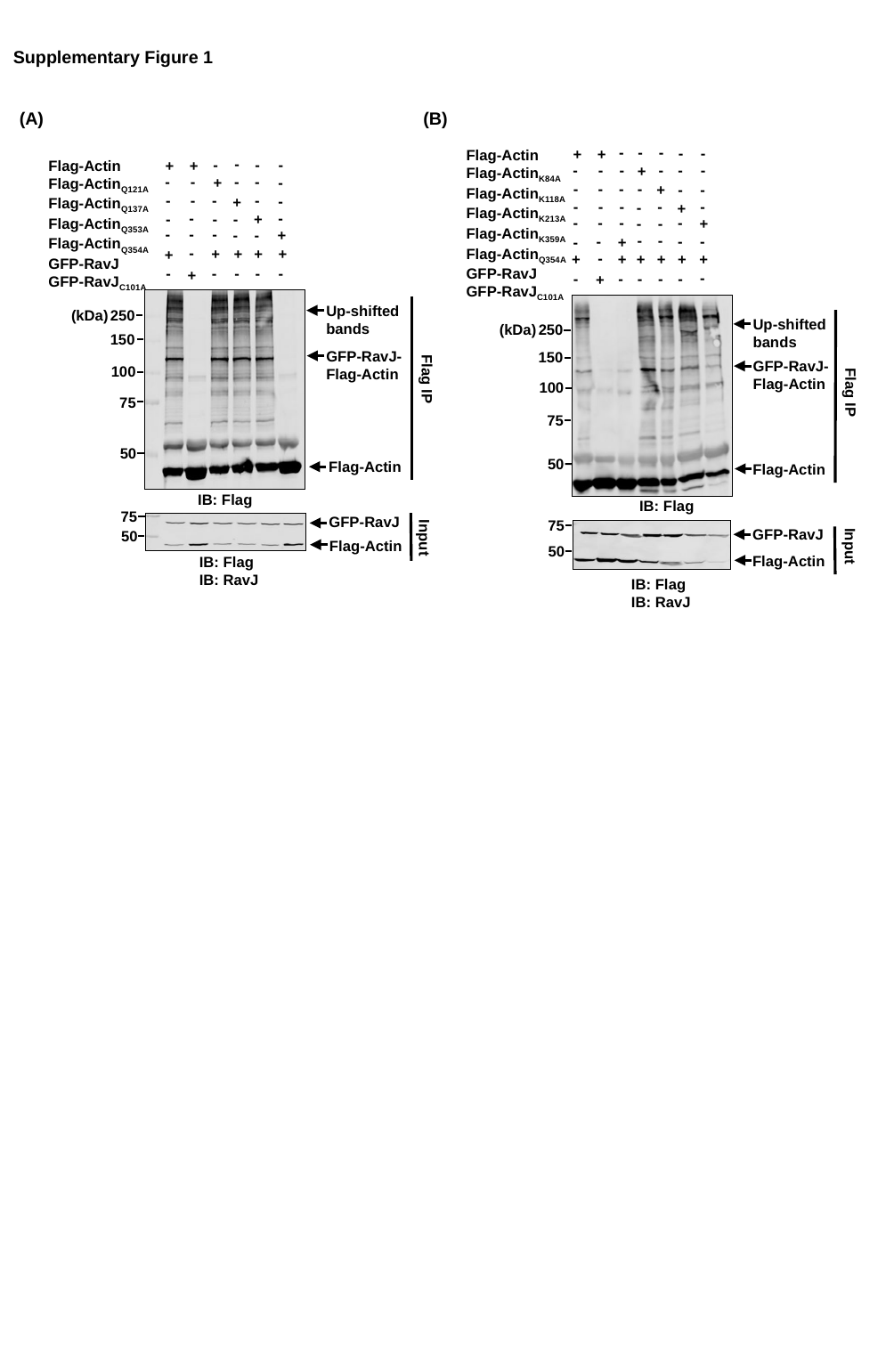

Supplementary Figure 1
(A)
(B)
-
-
-
-
-
+
+
Flag-Actin
Flag-ActinK84A
Flag-ActinK118A
Flag-ActinK213A
Flag-ActinK359A
Flag-ActinQ354A
GFP-RavJ
GFP-RavJC101A
-
-
-
-
Flag-Actin
Flag-ActinQ121A
Flag-ActinQ137A
Flag-ActinQ353A
Flag-ActinQ354A
GFP-RavJ
GFP-RavJC101A
+
+
-
-
-
-
-
-
+
-
-
-
-
-
+
-
-
-
-
-
-
+
-
-
-
-
-
+
-
-
-
-
-
-
+
-
-
-
-
-
+
-
-
-
-
-
-
+
-
-
-
-
-
+
-
-
-
-
-
-
+
-
+
+
+
+
+
-
+
+
+
+
+
+
-
-
-
-
-
+
-
-
-
-
-
-
+
Up-shifted bands
(kDa)
250
Up-shifted bands
(kDa)
250
150
GFP-RavJ-Flag-Actin
150
GFP-RavJ-Flag-Actin
100
Flag IP
100
Flag IP
75
75
50
50
Flag-Actin
Flag-Actin
IB: Flag
IB: Flag
75
GFP-RavJ
75
GFP-RavJ
50
Input
Flag-Actin
50
Input
Flag-Actin
IB: Flag
IB: RavJ
IB: Flag
IB: RavJ
